# The genome of *Pleurosigma* provides insights into the evolutionary adaptations of pelagic diatoms

**DOI:** 10.1093/dnares/dsaf037

**Published:** 2025-12-13

**Authors:** Jianbo Jian, Chunhai Chen, Xiaodong Fang, Christopher T Workman, Thomas Ostenfeld Larsen, Yuhang Li, Eva C Sonnenschein

**Affiliations:** Guangdong Provincial Key Laboratory of Marine Biotechnology, Shantou University, Shantou 515063, China; Department of Biotechnology and Biomedicine, Technical University of Denmark, 2800 Kongens Lyngby, Denmark; BGI Genomics, Shenzhen 518083, China; BGI Genomics, Shenzhen 518083, China; BGI Genomics, Shenzhen 518083, China; Department of Biotechnology and Biomedicine, Technical University of Denmark, 2800 Kongens Lyngby, Denmark; Department of Biotechnology and Biomedicine, Technical University of Denmark, 2800 Kongens Lyngby, Denmark; Laboratory of Marine Organism Taxonomy and Phylogeny, Qingdao Key Laboratory of Marine Biodiversity and Conservation, Institute of Oceanology, Chinese Academy of Sciences, Qingdao 266071, China; Department of Biotechnology and Biomedicine, Technical University of Denmark, 2800 Kongens Lyngby, Denmark; Department of Biosciences, Faculty of Science and Engineering, Swansea University, Swansea, SA2 8PP Wales, United Kingdom

**Keywords:** diatom, genomics, long-read sequencing

## Abstract

The diatom *Pleurosigma pacificum* is a newly described tropical pelagic species from the Western Pacific Ocean with one of largest genome size among published diatom genomes, making it an ideal candidate for studying adaptation to tropical open ocean environments and diatom evolution. We employed HiFi long-read sequencing to construct a high-quality and contaminant-free genome. The assembled genome is 1.357 Gb in size and consists of 821 contigs with a contig N50 of 3.23 Mb. The GC content is 38.6%, which is much lower than that of other published diatom genomes. The genome contains 27,408 predicted genes, 540 of which were implicated in environmental adaptation. Gene features and gene family comparisons suggest that the primary driver of genome expansion and functional diversification is long terminal repeats (LTR) retrotransposons and tandem duplications. The phylogenetic analysis revealed that the clade of *P. pacificum* is closely associated with other members of Naviculales. The expansion of chlorophyll a/c proteins might facilitate the adaptation of *P. pacificum* to high-light conditions in pelagic environments. The percentage of approximately 3.2% horizontal gene transfer (HGT) events is observed in the *P. pacificum* genome. HGTs are a prevalent phenomenon in diatoms and serve as a common mechanism to enhance their adaptive capabilities. In conclusion, the *P. pacificum* genome provides important understanding into the development of large genome size and evolutionary adaptations of pelagic diatoms.

## 1. Introduction

Diatoms (Bacillariophyta) are unicellular eukaryotic organisms that inhabit highly diverse aquatic environments, including freshwater and marine habitats as well as moist soils.^1^ As a highly diverse and successful group of ecologically important phytoplankton, diatoms contribute approximately 40% of the marine primary production and around 20% of the global carbon fixation.^2^ Recent gene-marker based analyses using 16S rRNA and *psbO* have refined estimates of the relative contributions of diatoms and other phytoplankton groups to marine communities, without diminishing their recognised ecological importance.^3^ They play an indispensable role in sustaining marine life and performing crucial biogeochemical functions.^4^ Diatoms are considered one of the most diverse and ecologically significant groups of phytoplankton.^5^ They are believed to have originated from secondary and tertiary endosymbiosis, involving algal and heterotrophic ancestors and gene transfer from bacteria and other organisms.^6^ Comprehensive analysis of selected diatom genomes suggests that they possess a complex evolutionary history.^4,7^ Generally, diatoms are classified into two major groups based on their symmetry: centric diatoms (with circular and radial symmetry) and pennate diatoms (with elongated and bilateral symmetry).^8^ A distinguishing feature of diatoms is the intricate silica shell that encases their cells, known as the frustule. This complex and porous structure possesses unique properties, including providing structural support, facilitating gas exchange, determining diatom shape and size, acting as a photonic crystal, and representing species-specific diversity.^9^

Until now, 56 diatom genome assemblies have been published in the NCBI (National Center for Biotechnology Information) database.^10^ Thirty-four out of the 56 genomes were sequenced using short reads, resulting in fragmented genome assemblies. There are only seven genomes with a contig N50 greater than 1 Mb, while 40 genomes have a contig N50 less than 100 Kb. Also, only 11 annotations of these genomes are available in the NCBI database. Recently, 49 diatom species for which genome assemblies were previously unannotated have now been annotated.^11^ These data demonstrate that the field of diatom genomics is still relatively young and holds great potential for future research. However, encouragingly, the Joint Genome Institute (JGI) is currently undertaking a project to *de novo* assemble 100 diatom species (https://jgi.doe.gov/csp-2021-100-diatom-genomes/), with a focus on their function in capturing carbon dioxide and their aquatic diversity.

To date, there have been at least 18 published research articles on sequencing and analysis of nuclear diatom genomes, beginning with two model diatoms including the centric *Thalassiosira pseudonana*^12,13^ and pennate *Phaeodactylum tricornutum*,^13,14^ then followed by *Mayamaea pseudoterrestris*,^15^  *Thalassiosira oceanica*,^16^  *Fistulifera solaris*,^17,18^  *Synedra acus*,^19^  *Cyclotella cryptica*,^20^  *Fragilariopsis cylindrus*,^21^  *Pseudo*-*nitzschia multistriata*,^22^  *Seminavis robusta*,^23^  *Chaetoceros tenuissimus*,^24^  *Nitzschia* sp. Nitz4,^25^  *N. inconspicua*,^26^  *Skeletonema costatum*,^27^  *Plagiostriata* sp. CCMP470,^28^ and *Skeletonema marinoi*.^29^ The comparative genomics analysis revealed significant differences in genome structure and gene features between pennate and centric diatoms.^14^ According to the analysis of the genomic architecture of the commercial diatom species *N. inconspicua*, it was found that duplicated pathways for glycolysis, carbonic anhydrases, and fatty acid synthesis were expanded in a species-specific manner, which supports genetic mechanisms for biomass and bioproduct production.^26^ The unconventional genetic systems of diatoms displayed by the allodiploid genome structure may enhance biofuel production^17^ and genetic loci with divergent alleles adapted to extremely cold Southern Ocean environment^21^ as demonstrated for the environmental and industrial model system *F. cylindrus*. With the genomic analysis of the benthic diatom, *S. robusta*, it appears that high gene family expansions and tandem duplications may have played a central role in evolutionary adaptations to benthic habitats.^23^ These available genome sequences provide a valuable resource for investigating the evolutionary history, genomic diversity, adaptation mechanisms, and biomass/bioproduct production in diatoms.

Horizontal gene transfer (HGT) events, lineage-specific gene duplications, genome rearrangements, together with losses and pseudogenization, have facilitated the evolution of biological diversity.^30^ HGT is a key process of genomic evolution and diversification. It is a well-established phenomenon in prokaryotes, and it has been observed that approximately 1% of protist genes have undergone HGT events, resulting in lifestyle adaptations or survival strategies in highly variable environments.^31^ With phylogeny-based HGT analysis in nine sequenced diatoms, it was found that 3 to 5% of the diatom proteome was horizontally transferred from bacteria.^32^ HGT is one of the factors responsible for the chimeric nature of diatom genomes, which likely contributes to the heterogeneity of their physiological and ecological traits.^14^ HGT can occur via viruses and indeed diatom-infecting viruses have been identified in the genome of *Chaetoceros tenuissimus*.^24^ Also, hundreds of putative genes potentially originating from bacterial HGT events have been found in the genome of *P. tricornutum*.^14^ The bacterial origin of HGT genes involved in the biosynthetic pathway of cobalamin endow *F. cylindrus* with scavenging and adaptive capabilities.^32^ In *P. multistriata*, a total of 252 HGT genes were proposed to have a bacterial origin and play roles in energy metabolism, oxidative stress response, and substrate transport.^22^ While HGT occurs more frequently among bacteria, these examples demonstrate the importance of HGT in the evolution of diatoms.^33^

The diatom genus *Pleurosigma* (Pleurosigmataceae, Naviculales) is composed of euplanktonic, tychoplanktonic, and benthic diatoms. Currently, there are 114 recognized species in this genus according to the AlgaeBase database.^34,35^  *Pleurosigma* belongs to the raphid diatoms that have evolved a longitudinal slit, known as a raphe, throughout either the entire or partial valve face. Occurrence of the raphe enables benthic diatoms to have moving ability, which is one of the most significant evolutionary milestone for diatoms. In this study, we focused on the newly described species *Pleurosigma pacificum*.^36^  *Pleurosigma pacificum* inhabits the tropical pelagic environment, which is generally low in nutrients, particularly nitrogen and phosphorus, in comparison to coastal environments.^37^ Compared to other benthic and tychoplanktonic coastal *Pleurosigma* species, *P. pacificum* exhibits a lanceolate valve and raphe, an extremely thin shell, a rapid growth rate, and high-light resistance. These characteristics enable them to remain buoyant and maximize their exposure to sunlight for photosynthesis. To enhance comprehension of the evolution, adaptation, and HGT events in tropical pelagic diatoms, we investigated the genomic characteristics of *P. pacificum* and provide novel insights into the molecular mechanisms underlying the ecological adaptations to this specific environment.

## 2. Materials and methods

### 2.1. Sampling, genomic DNA extraction, library preparation, and sequencing

**Fig. 1.**
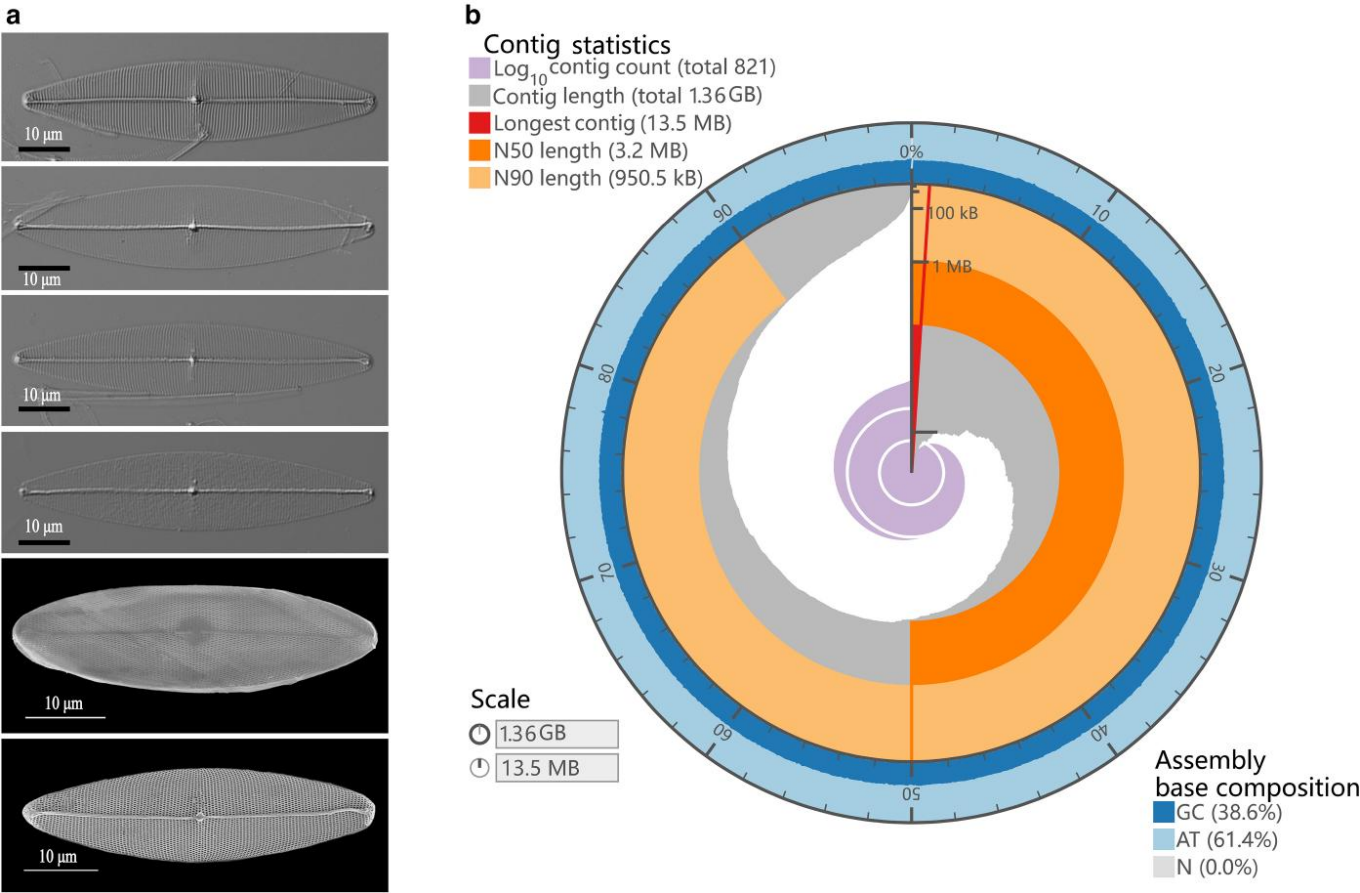
Images and genome characteristics of *P. pacificum*. a) Pictures of the single cell morphology of *P. pacificum.* b) Snail plot of genome assembly statistics including contigs, N50, N90, and GC content. The dark grey bars show record lengths in descending order, with the plot radius scaled to the longest record (red). The dark and pale orange arcs represent the N50 and N90 lengths, respectively. The pale grey spiral indicates cumulative record count on a logarithmic scale, with white lines marking each order of magnitude. The blue and pale-blue bands around the plot display GC, AT, and N percentages, using the same bins as the inner plot.

*Pleurosigma pacificum* samples were harvested from the upper 200 m water column using a phytoplankton net with a mesh size of 64 µm in the Western Pacific Ocean (7°0.26′ N; 141°59.63′ E) (Fig. 1a). Based on both morphology and molecular phylogeny, the strain was classified as a new species.^36^ The single cells of *P. pacificum* were isolated using capillary pipettes and subsequently cultivated in F/2 medium.^38^ The cultures were maintained at a temperature range of 24 to 26°C, with a photoperiod of 12:12 h (light/dark), under a light intensity ranging from 120 to 150 µmol m^−2^ s^−1^. To promote their growth, *P. pacificum* was cultured at 24 to 26°C under a light intensity of 200 µmol m^−2^ s^−1^, with a photoperiod of 14:10 (light/dark). To mitigate bacterial contamination, we incorporated antibiotics (30 U/ml Penicillin 30 μg/ml Streptomycin) into the culture medium and implemented microscopy to monitor the bacterial contamination. Sufficient biomass (∼5 g) was obtained for subsequent DNA extraction. A modified CTAB protocol was employed to extract high molecular weight genomic DNA.^39^

A short-insert (∼350 bp) genomic library of MGI-2000 sequencing was performed using MGIEasy PCR-Free DNA Library Prep Kit. The genomic DNA (∼3 μg) was physically fragmented using a Covaris S220 instrument (Covaris, Woburn, MA, United States). Subsequently, 100 to 200 ng aliquots of DNA fragments were size-selected to target size ∼350 bp and subjected to end repair and A-tailing. The experimental procedure involved sequential steps including adapter ligation, cleanup of adapter-ligated DNA, PCR amplification, and then, the library quality was evaluated by a Qubit and Agilent 2100 Bioanalyzer. Finally, the paired-end reads (PE 150) were sequenced using a MGISEQ-2000 sequencing platform. The low-quality reads, which include adapter sequences, PCR duplicates, N content (N bases >1%), or quality values ≤10 with low-quality base >20%, were removed by the software of SOAPnuke version 1.5.3.^40^ To generate a long insert (10 to 20 kb) PacBio library, a total of 10 µg purified diatom genomic DNA was utilized with PacBio SMRT Express Template Prep Kit 2.0 (Pacific Biosciences, CA, United States). Two SMRT Cells were sequenced using the PacBio Sequel II platform at the sequencing centre of Wuhan (BGI-Genomics, BGI-Shenzhen). The subreads obtained from sequencing were subjected to filtration using the SMRTLink (v8.0.0) circular consensus sequencing (CCS) algorithm with parameters ‘−minPasses 3 −minPredictedAccuracy 0.99 −minLength 500’ to generate high-quality reads.^41^

### 2.2. Genome survey, genome assembly, and genome curation

The genome size of the newly targeted *Pleurosigma* species is unknown, and the cost of long-reads sequencing is relatively high. To determine the optimal amount of data required for long-read sequencing, we first estimated the genome size based on short-read sequencing. The clean short reads data were utilized for 21-mer analysis, and jellyfish was employed to perform the profiling of 21-mer distribution.^42^ The genomic characteristics, including genome size, heterozygosity, and repeat content, were determined using Genomescope 2.0.^43^ A total of two SMRT Cells was sufficient for genome assembly, and the high continuous *Pleurosigma* genome was generated using hifiasm v0.7 with default parameters.^44^ To ensure accurate analysis, sequences derived from organelles or bacteria contaminants were removed to avoid confusion in HGT detection. All contigs were subjected to Megablast against the GenBank nucleotide (nt) database and Minimap2 mapping to the available algal organelle database. During the first decontamination step, the contigs, which received hits with >90% identity, aligned >500 bp, and a coverage >10% were considered as contaminants candidates. The assembled sequences were curated and subjected to contaminant removal based on bacterial and organelle alignment results, using GC content as a threshold (<33% or >56%). Then, based on the assembled genome V1, we conducted a second decontamination step by performing a MegaBLAST analysis against the NCBI Bacteria database^10^ with an e-value cutoff of 1e−5. The completeness of the assembled nuclear genome was evaluated using Benchmarking Universal Single-Copy Orthologs (BUSCO version 5.1.2) with the ‘eukaryote_odb10’ database that encompasses 255 conserved core eukaryotic genes.^45^

### 2.3. Genome annotation

The newly assembled nuclear genome of *P. pacificum* was utilized for repeat identification and gene annotation. The prediction of homologous repetitive sequences was initially performed using homology-based methods with LTR_FINDER v1.07^46^ (http://tlife.fudan.edu.cn/ltr_finder/) and RepeatModeler v2.0^47^ (http://www.repeatmasker.org/RepeatModeler/) based on the repeat database of RepBase v21.12 (http://www.girinst.org/repbase).^48^ Using *Ab Initio* method, we identified de novo repetitive sequences in the nuclear genome of *P. pacificum* through RepeatMasker 3.3.0.^49^ Subsequently, we employed three strategies, namely ab initio prediction, homology-based prediction, and RNA-Seq data-based prediction, to identify protein-coding genes in the *P. pacificum* genome. A total of 4.08 Gb of transcriptome data were sequenced from samples of *P. pacificum* and assisted in gene annotation. 27.2 million RNA-Seq reads were aligned with the newly assembled *P. pacificum* genome using hisat2.2.1,^50^ resulting in an overall alignment rate of 87.03%. The transcripts of *P. pacificum* were then identified by stringtie2.1.6^51^ based on the mapping result and applied to the next step of transcript-assisted annotation. The training parameter was applied to a set of 2,000 transcript-evidence genes. With the parameter trained, gene annotation was performed using ab initio prediction methods with AUGUSTUS v3.2.3^52^ and SNAP.^53^ In homology-based predication, seven available algal protein sequences from *F. cylindrus* CCMP1102 (GCA_001750085.1), *P. tricornutum* CCAP 1055/1 (GCF_000150955.2), *T. pseudonana* CCMP1335 (GCF_000149405.2), *S. robusta* (GCA_903772945.1), *C. tenuissimus* (GCA_021927905.1), *Chlamydomonas reinhardtii* v5.5 CC-503 cw92 mt + (GCA_000002595.3), *N. inconspicua* (GCA_019154785.2) were blasted to assembled genomes using TBLASTn.^54^ The gene set of *P. pacificum* were integrated using the MAKER pipeline (v3.31.8) with three types of evidence, including de novo, RNA-Seq, and homologue data.^55^ The predicted genes were functionally annotated using diamond (v0.9.10.111) blastp with an E-value threshold of ≤10^−5^ across seven databases, including TrEMBL, Swissprot, KEGG (Kyoto Encyclopedia of Genes and Genomes), KOG, InterPro, COG (Clusters of Orthologous Groups), and GO (gene ontology). The completeness of the gene set was assessed by BUSCO version 5.1.2 using the ‘eukaryote_odb10’ database for genome evaluation instead of the pep parameter.

### 2.4. Gene family and phylogenomic analysis

To determine the evolutionary clade of *P. pacificum* and conduct comparative analysis of gene families among diatoms, we utilized amino acid sequences derived from the genomes of *P. pacificum*, nine other diatoms species (*N. inconspicua*, *P. multistriata*, *F. cylindrus*, *P. tricornutum*, *S. robusta*, *T. oceanica*, *C. cryptica*, *T. pseudonana*, and *C. tenuissimus*), as well as one outgroup species, *Aureococcus anophagefferens*, to identify gene families. For the comparative genomic analysis across different diatom clades, we selected representative species with relatively high-quality genomes and annotations. Prior to clustering gene families, the gene sets of the 11 species underwent processing that involved filtering out genes with protein lengths less than 50 amino acids and retaining only the longest transcript. In total, 221,765 protein-coding genes were subjected to an all-against-all similarity search using Diamond (v0.9.10.111) with an e-value cutoff e10^−5^. Orthogroup clustering of all the target genomes was performed using OrthoFinder-2.3.11,^56^ followed by inference of single copy homologous groups based on orthogroups with the MSA program. A total of 371 single copy orthogroups were extracted from the orthogroups clustering result and aligned using MAFFT (v7.310).^57^ The gappyout method was applied using trimAl (v1.4.1) software.^58^ A phylogenetic tree was performed with single-copy orthologous genes utilizing RAxML-8.2.11 (version8.2.12) with PROTGAMMALGX model.^59^ The outgroup species of *A*. *anophagefferens* was rooted using TreeBest (https://github.com/Ensembl/treebest). The divergence time was inferred through the model of MCMCtree in PAML (version 4.9j).^60^ Three calibrated divergence times, namely *A. anophagefferens–N. inconspicua* (138.0 to 583.3 Mya), *T. oceanica–P. tricornutum* (18.9 to 70.3 Mya), *P. tricornutum–N. inconspicua* (75.0 to 95.3 Mya), were utilized from the TimeTree database (http://www.timetree.org/) to calculate the divergence times. Based on the phylogenetic tree of 11 algae genomes, gene family expansion and contraction were analysed using the CAFE v4.2 pipeline.^61^ The resulting expanded or contracted gene families underwent KEGG and GO enrichment analysis to elucidate their functions. The KEGG enrichment analysis was performed with respect to a background set consisting of 9,920 functional KEGG genes. The protein data sets from the 11 selected algae were subjected to InterProScan analysis, and Pfam domains were utilized to compare differences among algal genomes. Principal component analysis (PCA) was utilized to demonstrate classification and difference based on the top 10% conserved Pfam domains, while a heatmap was generated to display the number of Pfam domains and their top 10 InterPro ID between *P. pacificum* and nine other diatoms.

### 2.5. Identification of horizontal gene transfer

In here, we focused on the HGTs between algae and bacteria and algae and viruses. The possible HGT events of the target genomes were identified with a modified HGTphyloDetect pipeline.^62^ Each protein sequence was queried against National Center for Biotechnology Information (NCBI) non-redundant (nr) protein database with diamond (v0.9.10.111).^63^ The super-kingdoms Bacteria, Virus, Archaea, and Eukaryota were designated as parameters for the outgroup and ingroup lineages within the clade of taxonomy (subphylum). The queries meeting the criteria of AI (Alien Index) > 45 and out_pct ≥ 90% have been identified as potential HGT candidates. Phylogenetic trees were constructed using the HGT candidate genes and nr proteins that matched each query sequence with hits (E-value <1e^−10^). The homologous protein sequences were subjected to multiple sequence alignment using MAFFT v7.310^57^ and trimmed with Trimal-1.4.1^58^ using the gappyout parameter. The topologies of the homologous genes were inferred using IQ-TREE multicore version 2.2.0.^64^ Finally, the phylogenies were rooted and visualized through iTOL v6^65^ and two targeted genes were manually assessed to determine the mode of HGT candidate proteins.

## 3. Results and discussion

### 3.1. Genome sequencing and assembly

To date, the genomic characterization of the *Pleurosigma* genus is primarily based on analyses of the mitochondrial and chloroplast genomes.^5,66,67^ To facilitate long-read sequencing strategies of also the nuclear genome, short reads were initially sequenced to estimate the genome size. The raw data from DNBseq were subjected to quality filtering to remove low-quality reads. A total of 52.6 gigabases (Gb) of short-insert size DNBseq data were generated, with Q20 and Q30 quality scores exceeding 97.5% and 93.3%, respectively (Table S1). With 21-mer analysis, a genome scope profiling revealed a two-peaked curve plot, indicating an estimated genome size of approximately 1.31 Gb and a heterozygosity rate of 2.56%. Additionally, it should be noted that the genome is predicted to be diploid (Fig. S1). The genome size of over 1.3 Gb was one of largest among sequenced diatoms, representing a more than 9-fold increase compared to the genome of *S. robusta*, which had a genome size of approximately 171 Mb.^20^ In general, a genome coverage of approximately 30× using PacBio HiFi sequencing is deemed suitable for genome assembly. For the assembly of *P. pacificum*, two cells were selected from the PacBio Sequel II platform, which generated approximately 30 Gb data per cell. The long-read length distribution revealed an enrichment of lengths between 10 and 25 k (Fig. S2). After filtering out sequences below 500 bp and performing CCS, two PacBio HiFi SMAT cells generated a total of 65.7 Gb (∼50× coverage) clean data from 4.3 M reads (Table S2). The data output from Cell 1 and Cell 2 was 37.1 and 28.6 Gb, respectively, with a read length N50 of 16,150 and 14,860 (Table S2). The initial genome assembly of *P. pacificum* comprised a total length of 1,403,309,249 bp with a contig N50 size of 3.19 Mb and consisted of 1,837 contigs generated by Hifiasm^44^ (Table S3). The size of the assembled genome is approximately 1.36 Gb, slightly larger than the estimated genome size. While bacterial contamination can be a challenge for algal genome assembly due to microalgae being maintained non-axenically in stock cultures in laboratories or culture collections,^68^ long reads have been demonstrated as an effective solution to avoid bacterial contaminants.^69,70^ Furthermore, HiFi long reads are extremely accurate, making the genome assembly more effective. After aligning contigs to the Nucleotide Database (NT) from NCBI^10^ and performing genome curation, we removed the any contaminated contig. A total of 946 contigs were removed; all but one were shorter than 145 kb, and 939 were under 50 kb. One specific contig with a length of 5,108,603 bp was found to be well-mapped for over 1.4 Mb with the genome of the bacterium of the genus *Muricauda*, which has previously been isolated from seawater samples in the West Pacific Ocean, further suggesting that it is a biological contaminant.^71^ Following genome curation, a total of 38,536,373 bp of contaminant sequences comprising 946 contigs were eliminated. Subsequently, the second decontamination step was performed based on the assembled genome V1. The mapped results showed sequence identity ranging from 77.28% to 100% and alignment lengths between 37 and 21,894 bp. All mapped regions in the assembled genome were analysed to calculate coverage. Using a 5% coverage threshold, we identified an additional 70 contigs, totalling 7,983,834 bp, as potential bacterial contamination. Consequently, 442 genes were excluded based on the V1 assembly decontamination. This resulted in a total of 1,356,789,042 bp with a contig N50 size of 3.23 Mb and comprised of 821 contigs (Table S1, Fig. 1b, and Table S4). The size and quality of the *P. pacificum* assembly surpasses that of most diatom genomes currently available, which typically have a genome size less than 180 Mb and contig N50 length less than 1 Mb, with the exceptions of *T. pseudonana* (1.27 Mb)^12^ and *N. inconspicua* (3.62 Mb).^26^ The newly assembled genome exhibited a completeness of 79.2%, with 73.7 single-copy BUSCOs and 5.5% duplicated BUSCOs identified in the ‘eukaryote_odb10’ database using BUSCO version 5.1.2^45^ (Table S5). The BUSCO completeness of the pennate *P. pacificum* genome was higher than that of other centric diatoms (with the highest being 76.1%) and comparable to that of other pennate diatoms (ranging from 76.1% to 80.8%), as previously reported.^72^ After gene annotation, the BUSCO evaluation showed that 93% and 75.3% of gene sets in the *P. pacificum* genome were identified in ‘stramenopiles_odb10’ and ‘eukaryote_odb10’ databases, respectively (Fig. S3), which is comparable with other diatoms,^26^ demonstrating a high quality of the genome assembly and gene annotation.

The *P. pacificum* genome exhibits relatively low GC content (38.6%) compared with the genomes of other diatoms (48.3% in *S. robusta*; 48.8% in *P. tricornutum*; 46.9% in *T. pseudonana*) and its GC distribution also exhibits a significant deviation from other diatoms (Fig. S4). The lower GC content is associated with an expanded repertoire of transposable elements (TEs) and may be associated with its ecological and evolutionary characteristics.^73^ The GC content of the *P. pacificum* genome may be attributed to the higher biochemical costs associated with GC base synthesis and nutrient limitations in pelagic environment as previously suggested for Chrysophyceae.^74^  *Pleurosigma* species are usually distributed in nutrient rich coastal area, however *P. pacificum* was isolated from tropical West Pacific Ocean. We think this genome of *P. pacificum* may indicate the adaptation mechanism of this species to tropical oligotrophic environments. This may not only be reflected in its large genome.

### 3.2. Repeat sequence and gene features

A total of 817.16 Mb of repeat sequences were identified using the RepeatMasker ab initio method, accounting for 60.23% of the genome (Fig. S5a and Table S6). When utilizing a repeat database, 91.2 and 206.7 Mb were annotated with TEs and TE proteins respectively, comprising 6.7% and 15.2% of the assembled genome (Fig. S5b and Table S6). After combining homologous and de novo TEs, a total of 57.65% TEs with a size of 782.17 Mb were annotated in the *P. pacificum* genome (Table S7). The percentage of repetitive sequences was highest in the published diatom genomes of *C. cryptica* (54%),^20^ followed by *C. tenuissimus* (53%)^72^ and *S. robusta* (22.6%).^23^ The dynamics of LTR evolution play a key role in driving changes in genome size. With a detailed classification of repetitive sequences, the analysis of retrotransposon subtypes revealed that LTRs (49.45%) are predominant in the *P. pacificum* genome, while DNA transposons are rare (Table S8). This suggest that LTR elements play a predominant role in driving evolutionary processes. The LTRs, which constitute most TEs, are prevalent in alga.^72^ In an evolutionary context, the ‘copy and paste’ mechanism of transposing in mobile genetic elements results in retrotransposons increasing their copy number when active. Their accumulation is a primary contributor to genome size expansion in higher eukaryotes, alongside polyploidy.^75^

**Fig. 2.**
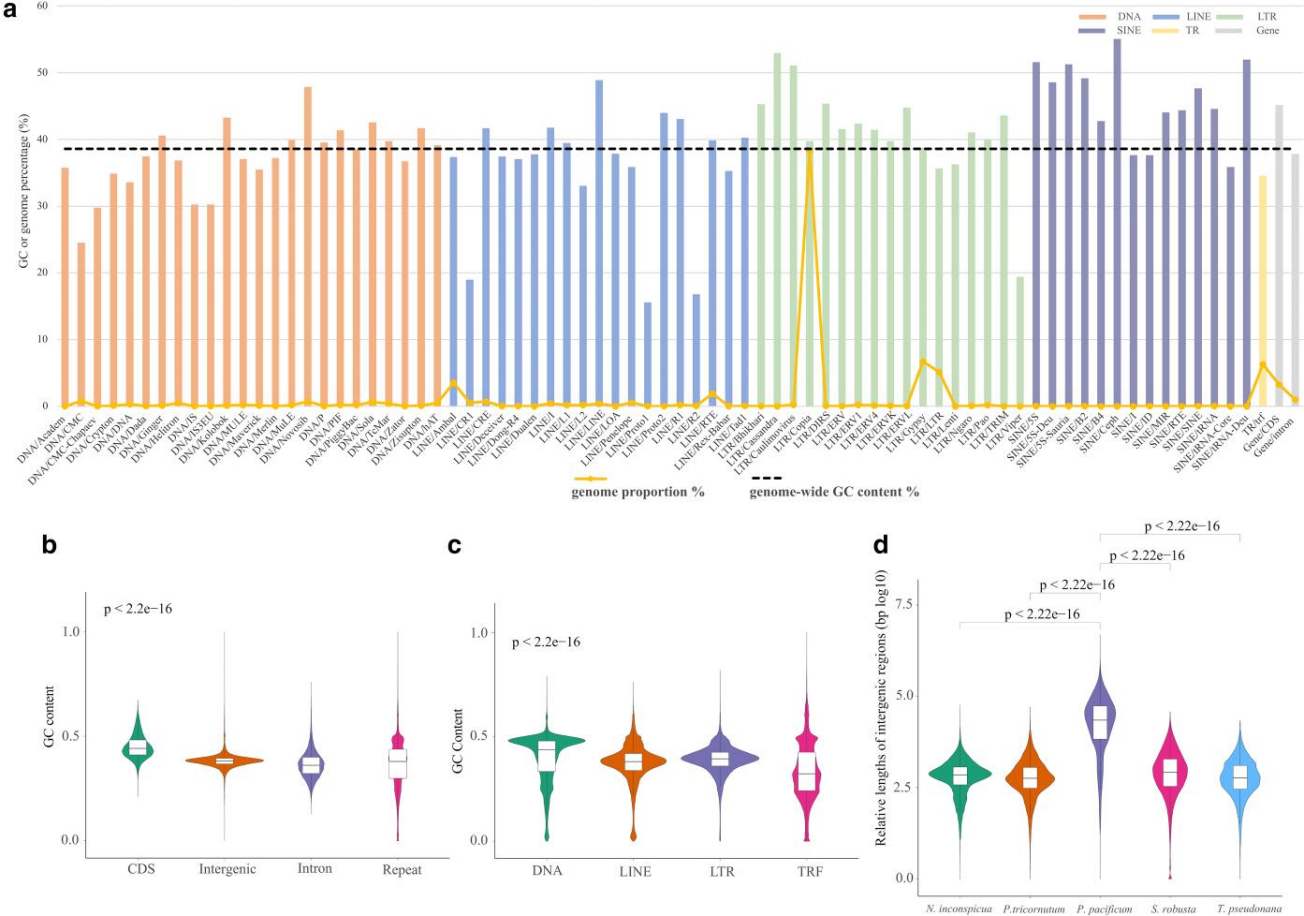
The statistics of GC content and comparison of intergenic regions of the *P. pacificum* genome. a) Histogram of the GC content and genome proportion for each subtype of repetitive sequences and genes. The height of the histogram column represents the GC content, while the yellow polyline indicates genome proportion and the dashed black line denotes average genome-wide GC content in % of DNA transposons (DNA), long interspersed nuclear elements (LINEs), long terminal repeats (LTRs), short interspersed nuclear elements (SINEs), and tandem repeats (TRs). b) The GC content of CDSs (coding sequence), intergenic regions, introns and repeats in *P. pacificum* genome (*P* < 2.2e^−16^ in intercomparions in Wilcoxon rank-sum test). c) The GC content in different types of repeats, namely DNA, LINE, LTR, and TRF in the *P. pacificum* genome (*P* < 2.2e^−16^ in intercomparions in Wilcoxon rank-sum test). d) Relative lengths (in bp) of intergenic regions in the genomes of *P. pacificum*, *N. inconspicua*, *P. tricornutum*, *S. robusta*, and *T. pseudonana* (*P* < 2.2e^−16^ in intercomparison to *P. pacificum* in Wilcoxon rank-sum test).

The analysis of GC and repeat contents indicated SINEs with high GC content of 45.8%, whereas Copia and Gypsy elements displayed a low GC content of 39.7% and 38.7%, respectively, and their average GC content was slightly lower at 38.6% (Fig. 2a). A large proportion of GC-poor DNA transposons contributed to the low GC content observed in the genome of *P. pacificum*. The TR/trf displayed a low GC content of 34.5%, whereas the gene/coding DNA sequences (CDS) exhibited a significantly higher GC content than other elements (Fig. 2b). The relatively high proportion of LINE/Ambal, TR/trf (tandem repeats**)** and LTR_LTR in the genome may contribute to its low GC content. The GC content shows a significant variation among the different types of repeats (Fig. 2c). The GC content of CDS was approximately 45%, which was significantly higher than the genome-wide average of 38.6% (Fig. 2b and Table S9). This phenomenon was also observed in nine other diatom species (Table S9). The expansion of GC-poor DNA transposons in *P. pacificum* may account for this phenomenon, as generating G–C requires more energy compared to A–T for *P. pacificum*. The genome size of 1.357 Gb represents the largest known genome in pennate diatoms to date, accompanied by the highest repeat content (60.23%). The proportion of repeat content ranges from 6.1% to 54% in the smallest diatom genome *T. pseudonana* (32.4 Mb) and eight other diatoms including *C. cryptica* (171.1 Mb) (Table S9). The high proportion of repetitive elements is just one factor contributing to the large genome size of *P. pacificum*, as repeats account for 54% of the genome below 200 Mb in size. Additionally, the coding sequence length of *P. pacificum* (44.9 Mb) is even smaller than that of *S. robusta* (54.1 Mb). The majority of *P. pacificum* sequences consist of intergenic regions, which are significantly longer than those found in other diatoms (Fig. 2d). The repetitive content accounts for 60.1% in a genome size of 1.36 Gb and 54% in genomes below 200 Mb. It appears that a potential turnover event may have occurred in algae, like the phenomenon observed in plant repeat turnovers.^76^ The higher proportion of LTR and tandem repeats may result in an increased genome size and a decreased GC content. After the identification of repeat sequences, a total of 27,408 protein-coding sequences (CDSs) were predicted. The number of genes in this species was significantly lower than that of *S. robusta* (37,718),^23^ but higher than other pennate diatoms (*P. tricornutum*: 10,409, *P. multistriata*: 11,895, *N. inconspicua*: 17,968, and *F. cylindrus*: 18,111).^14,21,22,26^ The significant variation may be attributed to differences in genome size, annotation methods, or species specificity. However, no positive correlation between genome size and number of CDSs has been identified in diatoms following this observed paradox.

The distributions of gene features in mRNA and CDS indicated that *P. pacificum* exhibited a similar pattern to *S. robusta*, *P. tricornutum*, *T. pseudonana*, and *N. inconspicua*, however, the plot for exon, intron, and exon number distribution showed a different pattern (Fig. S6). Notably, intron length is unique in *P. pacificum* with an average length of 588 bp compared to four other diatoms which range from 108 to 159 bp (Table 1 and Fig. S6). The increased length of introns in *Pleurosigma* may also serve as evidence that is influenced by environmental adaptation. The difference in gene features may support the diversity and adaptive evolution of algae.

**Table 1.** The genomics features of *P. pacificum* and four sequenced diatoms.

	*Pleurosigma pacificum* (this study)	*Seminavis robusta* (Sorokina et al. 2022)^27^	*Phaeodactylum tricornutum* (Bowler et al. 2008)^14^	*Thalassiosira pseudonana* (Armbrust et al. 2004)^12^	*Nitzschia inconspicua* (Oliver et al. 2021)^26^
Assembled genome size (Mb)	1,356,789,042	125,572,603	27,450,724	32,437,365	99,706,970
Sequencing technologies	PacBio sequel II	Illumina (main), PacBio	Sanger	Sanger	PacBio Sequel
No. of scaffold	891	4,752	88	64	125
Scaffold N50	3,228,431	50,704	945,026	1,992,434	3,618,388
No. of contig	821	4,960	179	115	125
Contig N50	3,228,431	48,501	417,209	1,267,198	3,618,388
GC content (%)	38.6	48.3	48.8	46.9	45.4
No. of genes	27,408	35,995	10,398	11,771	38,785
Gene average length (bp)	2,215	1,805	1,634	1,750	1,725
Intron average length (bp)	588	108	134	122	159

### 3.3. Functional characterization of the *P. pacificum* genome

The functional annotation of the *P. pacificum* genome revealed that 23,070 out of 27,408 genes (84.17%) were annotated using seven databases; however, only 39.39% could be annotated with the curated database Swissprot (Table S10). The level of annotation was slightly higher than for the diatom *C. weissflogii* (80%).^72^ However, only 59.8% (21,683 out of 36,254) of genes were functional annotated in *S. robusta*, indicating the potential existence of a larger number of uncharacterized genes awaiting annotation in this species.^23^ 36.19%, 36.08%, and 38.51% of the *P. pacificum* genes can be found in the KEGG, GO, and KOG databases, respectively (Table S10, Figs. S7 and S8). In three categories of GO (molecular function, biological process, and cellular component), 4,230 and 4,117 genes were found to be enriched in ‘metabolic process’ and ‘cellular process’, respectively, while 2,160 genes were enriched in ‘cellular anatomical entity’ (Fig. S7). The KEGG annotation revealed that most of genes were associated with metabolic processes, specifically 1,155, 588, and 515 genes involved in carbohydrate metabolism, amino acid metabolism, and lipid metabolism, respectively (Fig. S8). 338 and 181 genes were found to be enriched in the biosynthesis of various secondary metabolites as well as the metabolism of terpenoids and polyketides. Additionally, a total of 579 genes were implicated in environmental adaptation based on KEGG of functional annotations. These genes contribute to the understanding of pathways involved in nutrient assimilation, environmental adaptation, and diatom metabolism. With the aid of Venn diagrams depicting functional annotations in Nr, Swissport, KEGG, KOG, and InterPro databases, a total of 8,549 genes were identified across all five databases (Fig. S9). Additionally, 5,308 and 380 genes were only annotated in NR and InterPro, respectively, suggesting that a significant number of genes is not well described across databases.

### 3.4. Functional genomic comparison

The InterProScan tool was utilized to identify InterPro domains in eleven algal genomes. PCA was applied to the InterPro annotation data of the top 10% most frequent domains to investigate patterns of functional diversity.^77^ Based on PCA1 vs. PCA2, InterPro domains revealed that Naviculales group together with Bacillariales and *T. pseudonana*, while the other Thalassiosirales and the Chaetocerotanae species lie separately (Fig. S10). The genome of *P. pacificum* genome cloud be distinguished from those of other species through PCA3 vs. PCA4 analysis, this analysis also identified specific InterPro domains that were unique to *P. pacificum* (Fig. S10). The PCA results may suggest that species within the same cluster have undergone similar evolutionary changes associated with functional Pfam domains. *Pleurosigma pacificum*, which possessed the largest genome size and the highest gene count, also displayed a significantly greater number of independently annotated InterPro domains, as demonstrated by the PCA analysis of these domains. The heatmap of Pfam (top 10% most abundant annotated domains in all 10 genomes) was generated using a total of 569 domains for clustering analysis (Fig. S11). The number of domains identified in *P. pacificum* was higher than that observed in other species and exhibited a distinct pattern compared to the other ten algal species, with the most similar heatmap pattern observed in *S. robusta* (Fig. S11). Comparing the top 10 InterPro domains of *P. pacificum* and nine other diatom species, the domain IPR005046 (domain of unknown function 285) showed the greatest difference compared to its abundance in the genomes of the other nine diatom species, followed by IPR001846 (vWDs: von Willebrand factor, type D domain) (Fig. S12). The vWDs domain is a highly conserved protein motif that plays a crucial role in mediating the formation of multiprotein complexes through adhesive interactions.^78,79^ The greater abundance of the vWDs family indicates multiprotein complexes play an important role in the adaptation to open sea environments as well. Notably, the IPR000719 domain, which is involved in protein kinase function (protein phosphorylation), ranked among the top 10 InterPro domains of *P. pacificum* and was identified in over 124 genes in each diatom. This component plays a key role in cellular activities. However, its prevalence was significantly higher in *P. pacificum* and *S. robusta* (Fig. S12). Three domains, namely IPR003593, IPR018247, and IPR008271, were found in more than 230 genes of *P. pacificum*, but only present in two or fewer genes of the other nine diatoms examined. These three domains are involved in the function of ATP hydrolysis activity, calcium-binding, and protein phosphorylation, which are unique to *P. pacificum* and potentially play a crucial role in its evolution.^80–82^ IPR001846 (vWDs) and IPR001767 (Hedgehog protein, Hint domain) were detected in over 290 genes of *P. pacificum* but showed minimal occurrence in the nine diatoms except for *S. robusta* with a count of 20 and 112, respectively (Fig. S12). The Hint domain of the Hedgehog protein has been identified in sub-telomeric gene duplications, indicating its role in the adaptation of red algae to extreme environments.^69^

### 3.5. Phylogenetic analysis of *P. pacificum*

**Fig. 3.**
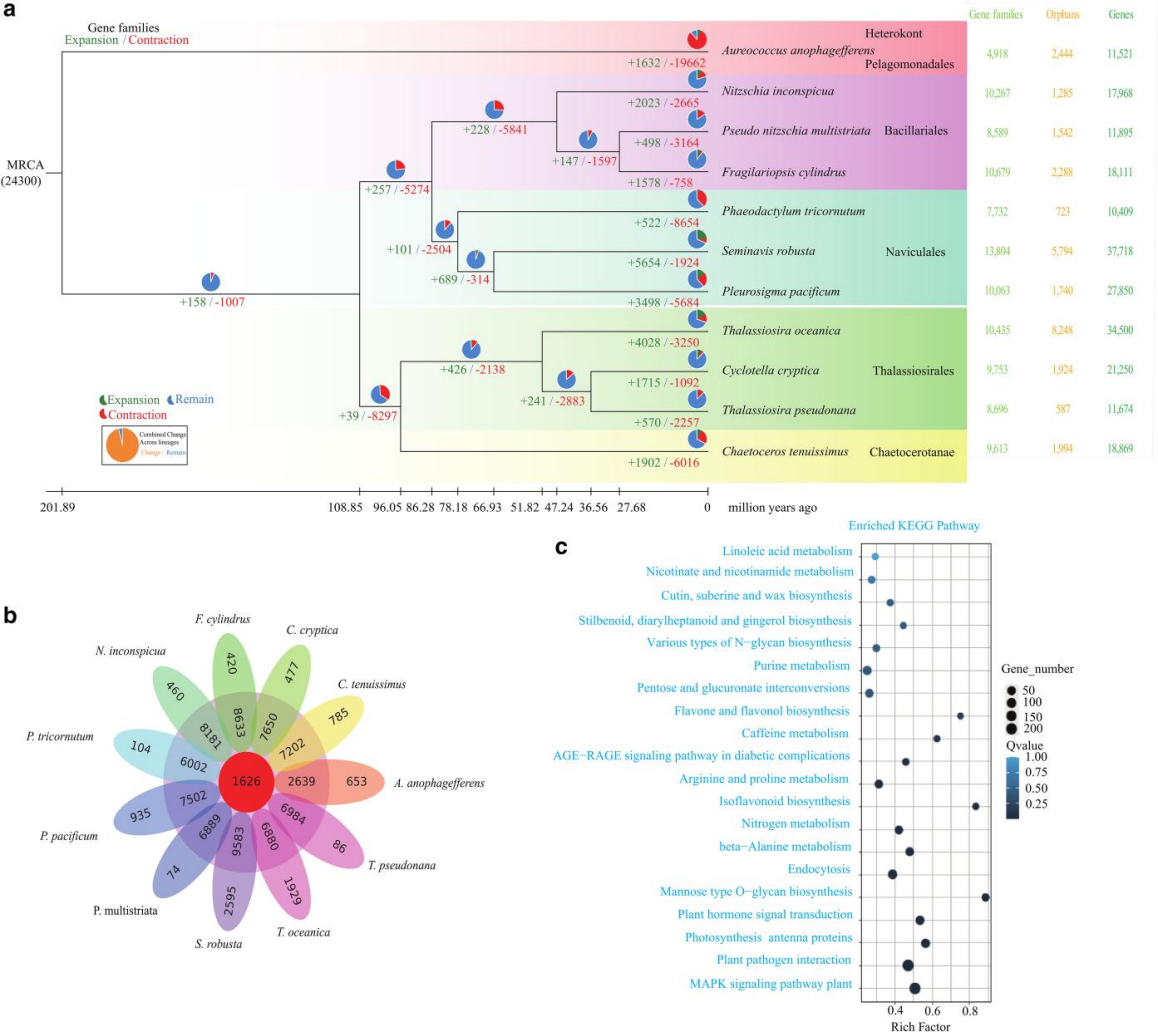
Comparative genomic analysis of diatoms. a) The phylogenetic tree of expanded and contracted gene families for *P. pacificum* and other diatoms and total number of gene families, orphans, and genes per diatom genome. b) The Venn diagram of shared and unique gene families in *P. pacificum* and 10 other diatom species. c) The KEGG enrichment of expanded gene families in the genome of *P. pacificum*. The Rich factor is the ratio of enriched gene numbers annotated in this pathway to all gene numbers annotated in this pathway.

An accurate phylogenetic tree enhances our comprehension of clade and evolutionary transitions in diatoms. Based on the SSU rDNA and rbcL sequences, *P. pacificum* was positioned in a basal position relative to other *Pleurosigma* species.^36^ The clade of Pleurosigmataceae including *Pleurosigma*, *Rhoicosigma*, *Carinasigma*, *Donkinia*, and *Gyrosigma*, is sister to the clade of *Navicula* and *Haslea*. This phylogenetic relationship also supported by a mitochondrial phylogeny.^67^ The protein-coding genes of *P. pacificum* and nine representative diatom species and the heterokont outgroup *A. anophagefferens* were clustered using OrthoFinder-2.3.11.^56^ The number of genes in orthogroups ranged from 9,077 (*A. anophagefferens*) to 31,924 (*S. robusta*), with the highest percentage of genes in orthogroups being 95% in *T. pseudonana* and the lowest being 76.1% in *T. oceanica* (Fig. S13). In *T. oceanica*, the number of genes in species-specific orthogroups is 10,745, accounting for 31.1%, which is followed by *S. robusta* (28.3%) and *P. pacificum* (23%) (Fig. S13). In the species-specific family results, the presence of a higher number of protein-coding genes in *T. oceanica*, *S. robusta*, and *P. pacificum* indicates an increased abundance of gene families compared to the lower number observed in *T. pseudonana*, *P. multistriata*, and *P. tricornutum*, which also have smaller genome sizes (Fig. S13). In total, 1,626 orthogroups were identified across all 11 algae species and a corresponding species tree was constructed (Fig. 3a and b). Notably, the same tree topology was observed when analysing both single copy orthogroups (Fig. S14) and common orthogroups (Fig. 3a). The phylogenetic tree analysis revealed that the three species of each sub-groups belonging to Bacillariales, Naviculales, and Thalassiosirales formed a monophyletic clade consistent with their taxonomic classification at the family level (Figs. 3a and S14). *Chaetoceros* is classified as an outgroup of the Thalassiosirales within centric diatoms. *Pleurosigma pacificum* is most closely related to *S. robusta*, and centric (Thalassiosirales, Chaetocerotanae) and pennate (Bacillariales, Naviculales) diatoms are clearly separated into two clades (Figs. 3a and S14). The phylogenetic relationships derived from the diatom genomes are consistent with previous reports.^23,72^ The divergence time was estimated based on three calibrated times from a time tree. The divergence time of centric diatoms and pennate diatoms was about 108.9 million years ago (Ma). The split between the family of Bacillariales and Naviculales occurred 86.3 Ma. The divergence time between *P. pacificum* and benthic diatoms *S. robusta* was approximately 66.9 Ma, which occurred around 78.2 Ma after their divergence from *P. tricornutum* (Fig. 3a).

### 3.6. Comparative analysis of transcription-associated proteins

Both transcription factors (TFs) and transcriptional regulators (TRs) are two classes of proteins that play crucial roles in regulating gene expression. Additionally, protein kinases (PKs), which constitute a large family of enzymes, play critical roles in cellular signalling and regulation. No significant differences were observed in the number of TFs, TRs, and PKs between pennate and centric diatoms in this study (Figs. S15–S17). Compared with eight other diatoms, few TF families are expanded in *P. pacificum* (Fig. S15). Notable exceptions are cold shock domain (CSD) TFs, which are one of the most evolutionarily conserved proteins.^83–85^ Twenty CSD genes were identified, whereas all other eight diatoms encode between five and nine (Fig. S15). Nine CSD domains were identified in the cold-adapted diatom *F. cylindrus*,^21^ which was significantly fewer than those found in *P. pacificum*, indicating that CSD played more important roles in both developmental processes and stress responses. *Pleurosigma pacificum* and *S. robusta* exhibited an expansion of bZIP (basic leucine zipper) TFs, with 26 and 31 genes, respectively, which facilitate colonization of new environments.^86,87^ The TR gene families in *P. pacificum* were significantly expanded, with a total of more than 500 TR genes, whereas all other diatoms encode less than 300, except *S. robusta* with 556 (Fig. S16). The main expanded TR is high mobility group (HMG) with a total number of 238, while all other diatoms carry below 35 HMG TRs (Fig. S16). The HMG proteins, which can be classified into three families (HMGA, HMGB, and HMGN) ,^88^ are major components of chromatin architecture in eukaryotes and play a multifaceted role in regulating chromatin dynamics.^89,90^ The majority of 238 HMG gene loci exhibited co-localization with tandem repeats. The PKs genes were significantly expanded in both *P. pacificum* and *S. robusta* with the number of 395 and 414, respectively, while the other six diatoms had 129 to 218 PKs genes (Fig. S17). Among these expanded PKs in *P. pacificum* were four types of PKs groups: 138 CDPKs (calcium-dependent protein kinases), 26 WNK (with-no-lysine kinase), 17 DCAMKLs (CaM kinase-like), and 24 AGC_PKA-PKG (protein kinase A, G, and C, cAMP and cGMP-dependent protein kinase). The expanded PKs were tandemly duplicated kinase genes. These PKs may have participated in evolutionary adaptations, while the CDPKs may function as survival mechanisms under adverse environmental conditions.^91^  *Pleurosigma pacificum*’s euplanktonic nature in the open ocean may have enabled external stimuli (such as light) to modulate AGC kinase activity.^92^

### 3.7. Gene family expansions in *P. pacificum*

To elucidate the evolutionary and adaptive mechanisms of diatoms, we conducted gene family expansions and contractions analysis, revealing 3,498 expanded gene families and 5,684 contracted gene families in *P. pacificum* (Fig. 3a). The KEGG enrichment analysis of 488 expanded gene families in *P. pacificum*, with a significance level of *P* < 0.05, revealed significant enrichments (Qvalue <0.01) in the Mitogen-activated protein kinase (MAPK) signalling pathway (224), plant–pathogen interaction (242), photosynthesis-antenna proteins (67), plant hormone signal transduction (64), mannose type O-glycan biosynthesis (23), endocytosis (82), beta-alanine metabolism (96), and nitrogen metabolism (37) (Fig. 3c). The MAPK families play a crucial role in the regulation of cell cycle progression and stress responses. While ubiquitously present in algae, green algae exhibit a relatively low number (2 to 5) of characteristic MAPKs across 13 representative green algal species.^93^ The expanded gene family implicated in plant–pathogen interactions provides compelling evidence for the coexistence of diatoms and bacteria in common habitats over an extensive evolutionary timeframe spanning hundreds of millions of years.^94^ The diatoms are typically dominant competitors and exhibit relatively restricted ecological interactions with other marine eukaryotes.^95,96^ The interaction between diatoms and marine microbial communities in the ocean play essential roles to this ecosystem.^94,97,98^ The enrichment of signalling pathways may contribute to the specific interactions within the phycosphere.^94^ These interactions could contribute to the ability of *P. pacificum* to its competitive advantage over other organisms under eutrophic conditions, and its roles in plankton communities and evolution.^99^ The same gene families that were expanded, as identified by KEGG enrichment analysis, including those involved in endocytosis and photosynthesis-antenna protein, were also observed in the diatom *Skeletonema marinoi*.^29^ To adapt to the strong light conditions in Western Pacific Ocean for *P. pacificum*, the pelagic diatoms maybe dissipate excess energy through the photosynthesis-antenna protein.^100^ The photosynthetic antenna proteins efficiently utilize light energy in high-light environments on the ocean surface.^100,101^ The genome of *P. pacificum* contains a total of 128 genes encoding chlorophyll a/c proteins (IPR022796) as antenna, which is the highest number among diatom genomes studied to date. In comparison, *S. marinoi* has 100 such genes,^29^  *S. robusta* has 83, and other species exhibit fewer than 71. The structural and spectroscopic properties of fucoxanthin chlorophyll (Chl) a/c-binding proteins (FCPs) further enhance the ability of diatoms to adapt to fluctuating light environments.^102^ The largest number of chlorophyll a/c proteins may potentially facilitate the adaptation of *P. pacificum* to high-light conditions in pelagic environments. Only four genes exhibited a significant (*P*≤0.05) contraction, which were involved in NLRC3 (NLR Family CARD Domain Containing 3), which are immune-related genes.

The NLRC3 protein, a member of the NLR family of cytosolic pathogen recognition receptors, may contribute to an increased susceptibility for horizontal gene transfer due to its contraction in *P. pacificum*. The KEGG enrichment of expanded gene in the clade of *P. pacificum* and *S. robusta* showed the same in MAPK signalling pathway, Plant hormone signal transduction, plant–pathogen interaction, and nitrogen metabolism (Fig. S18). The gene family cluster comparison in diatoms revealed that *S. robusta* had the highest number of 493 gene families, followed by *F. cylindrus* with 217, and *N. inconspicua* with 198. Analysing the intersection of gene families among *P. pacificum* (343), *S. robusta* (340), and *T. oceanica* (292) (Fig. 3b) showed that *P. pacificum* exhibited a similar genomic evolution not only in phylogenetic relation but also in gene expansion.

### 3.8. Horizontal gene transfer in diatoms

**Fig. 4.**
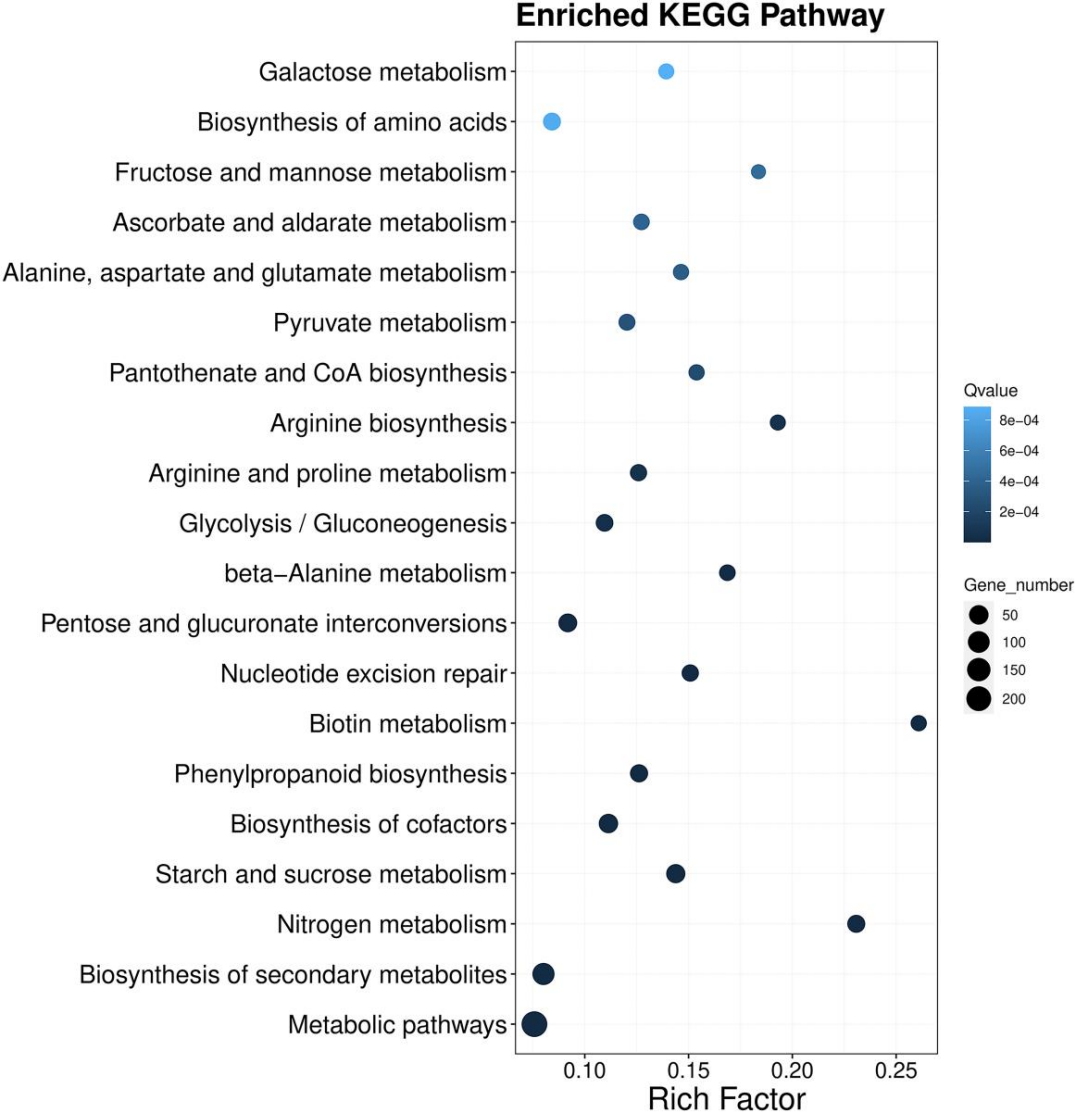
Enriched KEGG pathways of putative HGT genes in the *P. pacificum* genome.

**Fig. 5.**
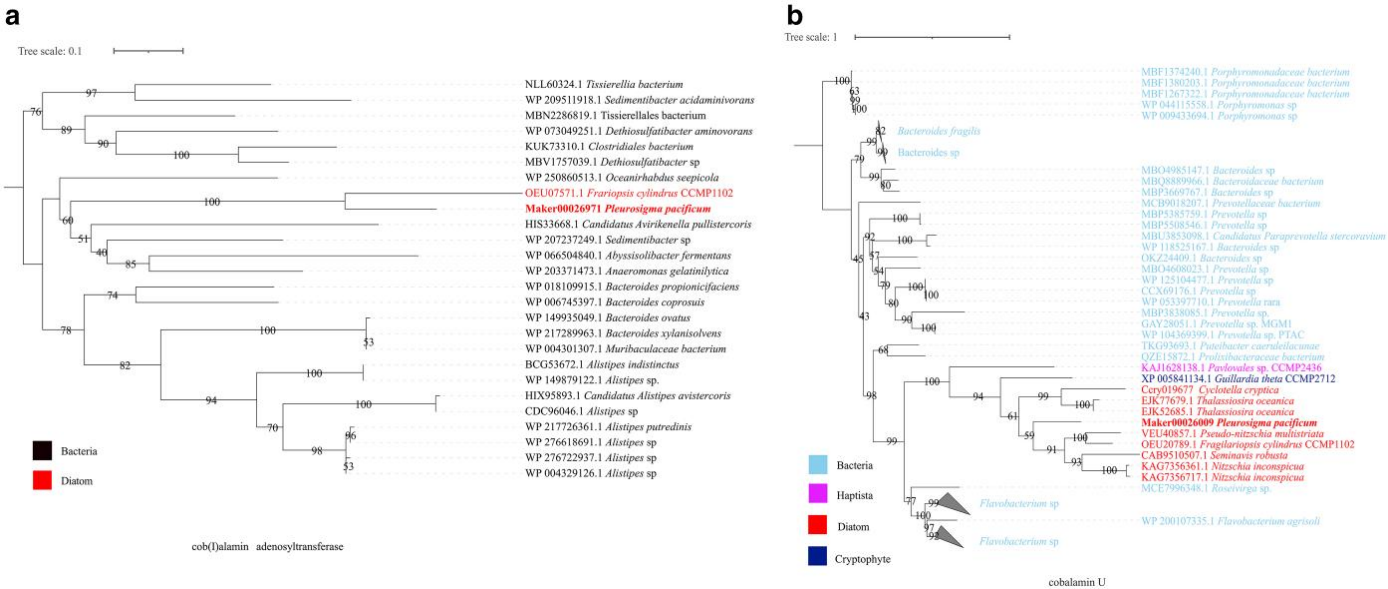
Phylogenetic trees of two cobalamin genes putatively obtained by HGT. a) The phylogenetic tree of HGT cob(I)alamin adenosyltransferase. Bacteria are marked in black, diatoms are marked in red. b) The phylogenetic tree of the HGT cobalamin U gene. A maximum-likelihood IQ-TREE with 1,000 bootstraps was performed using putatively horizontally transferred genes, along with aligned bacterial and algal genes from the nr databases. Bacteria are marked in light blue, Cryptophyte in dark blue, haptista in pink and diatoms in red.

Putative HGT events, potentially occurring from prokaryotes, were investigated in the ten diatom species. A total of 8,511 diatom genes were identified as putative HGT events from prokaryotes, covering 770 gene families (orthogroups).^56^ A total of 513 to 1,724 HGT candidate genes per diatom species were identified using HGTphyloDetect pipeline prior to constructing a phylogenetic tree (Table S11). Each HGT percentage was higher than 3% except in *T. oceanica* (2.1%). The HGT percentage was less than 5% in 7 of 10 diatoms (∼3.22% in *P. pacificum*), except 5.79% in *C. cryptica* and 5.76% in *T. pseudonana* (Table S11). The percentages were comparable to those previously reported in *Blastocystis hominis* (2.5%)^103^ and *P. multistriata* (3.6%),^22^ but lower than in *P. tricornutum* (4.8%),^14^ in the red alga *Porphyridium purpureum* (5.4 to 9.3%)^104^ and in nine other diatoms.^32^ HGT was identified as a prevalent mechanism utilized by diatoms to facilitate their adaptive capabilities.^32^ In the 882 putative HGT genes identified in *P. pacificum* excluding those with unknown taxonomy, a total of 53 genes were annotated as being involved in oxidoreductase activity (GO:0016491), followed by 27 with ATP binding (GO:0005524) and 21 associated with membrane (GO:0016020). Therefore, *P. pacificum* may adapt to cope with strong light stress by undergoing oxidoreductase. The enrichment of KEGG pathways revealed significant enrichment of HGT genes in various pathways such as biosynthesis of secondary metabolites, galactose metabolism, pantothenate, and CoA biosynthesis and biosynthesis of amino acid (Fig. 4). In the open western Pacific Ocean, *P. pacificum* should face limited nutrition comparing to benthic environments. Urea metabolism has been demonstrated to facilitate diatom recovery from prolonged nitrogen limitation by fixing carbon into nitrogenous compounds.^105^ Two related genes (carbamate kinase and ornithine cyclodeaminase) in this pathway have been identified as laterally transferred in previous studies.^14,105^ For cobalamin (vitamin B_12_), a well-known nutrient whose biosynthesis is lacking in more than half of the algae including diatoms,^106^ its availability has been found to be dependent on bacteria.^107^ Methionine synthase, methylmalonyl-CoA mutase and type II ribonucleotide reductase (RNRII) are three vitamin B_12_-dendent enzymes in eukaryotes. Regarding ATP:cob(I)alamin adenosyltransferase, this is enzyme is involved in the stabilization of (likely externally acquired) B_12_ as a cofactor, but not its synthesis. This, nor the fact that it was identified as HGT. In *P. pacificum*, which cannot produce its own vitamin B12, a putative gene was identified, including ATP:cob(I)alamin adenosyltransferase (Maker00026971) (Fig. 5a). This gene may be crucial for *P. pacificum* to enhance its adaptability to the open ocean habitat. Similarly, the cobalamin-related gene (*bluB*) in *F. cylindrus*, *P. multistriata*, and *P. multiseries* originated by HGT from alphaproteobacteria.^32^ In this study, however the Cobalamin U (CobU) gene with an AI value of 59.5 was filtered out with an out_pct ≥ 90% due to the out_pct below 90%. With the consideration of out_pct ≥ 85%, the CobU HGT was identified in seven diatoms including *C. cryptica*, *F. cylindrus*, *N. inconspicua*, *P. pacificum*, *P. multistriata*, *S. robusta*, and *T. oceanica* (Fig. 5b). Furthermore, also *Pavlovales* sp. CCMP2436 was found to have benefited from HGT of *cobU*. The *cobU* gene identified in eight diatoms was grouped into a single clade, indicating that the HGT events occurred in the ancestor of diatoms.^32^

## 4. Conclusions

*Pleurosigma pacificum* is a planktonic species newly collected from the surface water of open Western Pacific Ocean. Since most *Pleurosigma* species are benthic and tychoplanktonic living in coastal environments, *P. pacificum* may have a benthic origin. *Pleurosigma pacificum* carries the largest known diatom genome indicating unique adaptations to the transition from low light and high nutrient sediment to the high-light and low-nutrient open ocean. The genome of *P. pacificum* with 1.36 Gb sequences was assembled based on HiFi long reads. The genome exhibited high heterozygosity (2.5%) and was assembled into high quality with a contig N50 size of 3.23 Mb. The repeat sequences accounted for 60.23% of the genome, and the low GC content (38.6%) observed in *P. pacificum* was largely attributed to GC-poor DNA transposons. The gene features and InterPro domains showed that the *P. pacificum* genome has specific characteristics compared to other diatoms. The expanded transcription factors, transcriptional regulators and protein kinases were produced by tandem duplicated repeat expansion. The expanded gene family were enriched significantly in plant–pathogen interaction, MAPK signalling pathway, endocytosis and more, indicating the adaptation to open sea environments. Nearly 3.2% of genes were identified to be putatively derived by HGT from prokaryotes including cobalamin and oxidation–reduction related genes. The HGT genes were enriched in various pathways, including biosynthesis of secondary metabolites, pantothenate and CoA biosynthesis, and amino acid biosynthesis, which could have occurred in response to high-light and nutrient limitation. The high-quality *P. pacificum* genome will be a valuable resource for ecological, adaptation studies of diatoms and resolving HGT events in algae.

## Supplementary Material

dsaf037_Supplementary_Data

## Data Availability

The data that support the findings of this study have been deposited into CNGB Sequence Archive (CNSA) of China National GeneBank DataBase (CNGBdb)^108^ with accession number CNP0004331 and at NCBI under the project PRJNA1150279. The genome sequence accession ID is CNA0069027.
