# Supplementary material for: The genome of Pleurosigma provides insights into the evolutionary adaptations of pelagic diatoms
Source: DNA Res. 2025 Dec 13;33(1):dsaf037. doi: 10.1093/dnares/dsaf037 (PMC12803021; doi:10.1093/dnares/dsaf037)
Supplement: dsaf037_Supplementary_Data [file dsaf037_supplementary_data.docx]

Supplementary materials

**The genome of *Pleurosigma* provides insights into the evolutionary adaptations of pelagic diatoms**

Jianbo Jian, Chunhai Chen, Xiaodong Fang, Christopher T. Workman, Thomas Ostenfeld Larsen, Yuhang Li, Eva C. Sonnenschein

# Figure S1. Genome survey of *Pleurosigma pacificum* using GenomeScope.


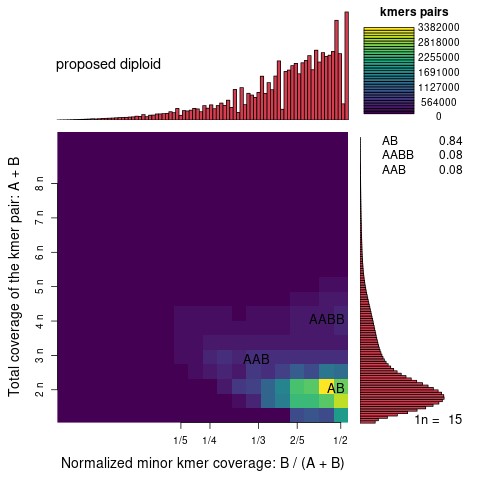

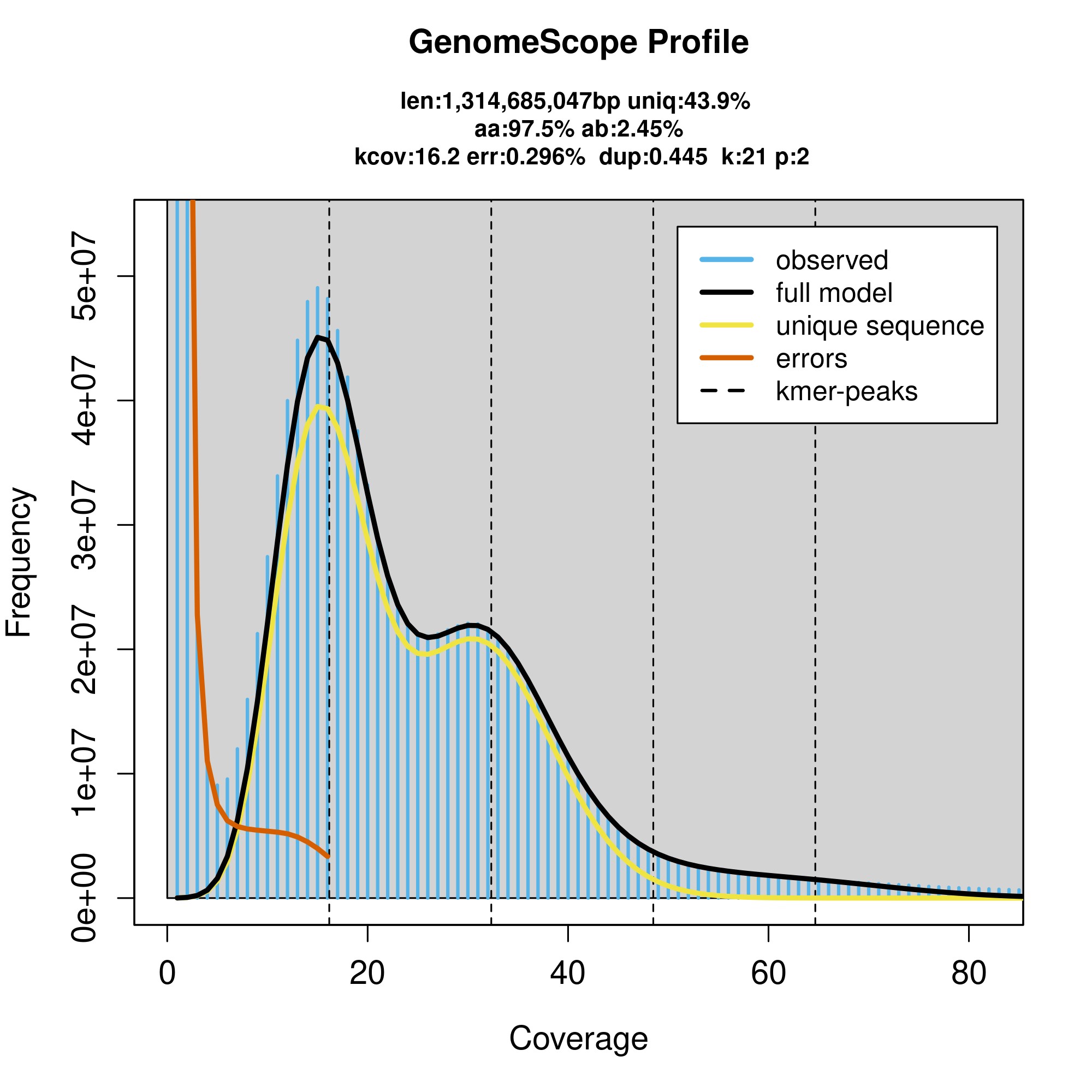


X axis is the coverage (X), y axes is the frequency of 21-mers.

Note: het: heterozygosity; kcov: kmer mean peak; uniq: non-repetitive kmer; observed: the kmer actual distribution by jellyfish analysis; full model: theoretical kmer distribution; unique sequence: non-repetitive kmer; errors: wrong kmer, usually the lower kmer；kmer-peaks: the position of the kmer peak.

# Figure S2. The length distribution of the HiFi sequencing subreads.


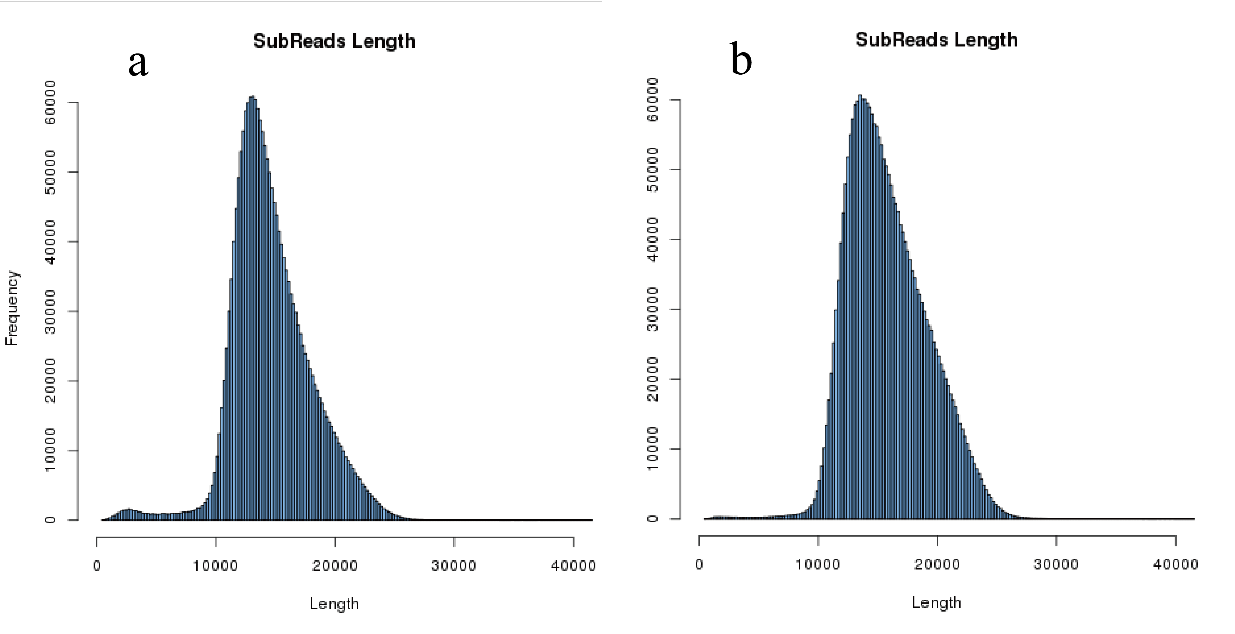


a: The subreads of Cell 1. b: The subreads of Cell 2.

# Figure S3. BUSCO evaluation of *P. pacificum* and other diatom with two databases eukaryota_odb10 and stramenopiles_odb10.


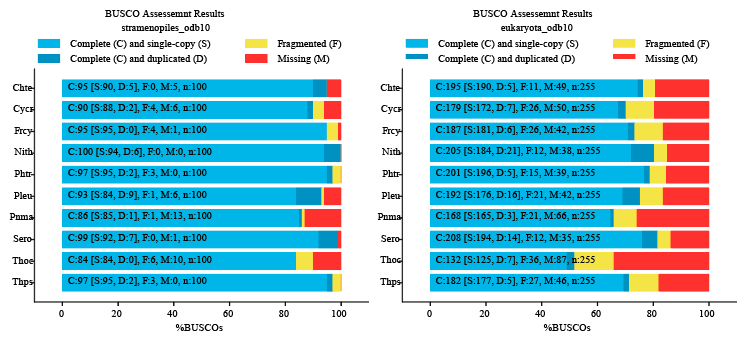


*(Chaetoceros tenuissimus*: Chte; *Cyclotella cryptica*: Cycr; *Thalassiosira oceanica*: Thoc; *Thalassiosira pseudonana*: Thps; *Fragilariopsis cylindrus*: Frcy; *Nitzschia inconspicua*: Nith; *Phaeodactylum tricornutum*: Phtr; *Pleurosigma pacificum*: Pleu; *Pseudo nitzschia multistriata*: Pnma; *Seminavis robusta*: Sero)

# Figure S4. The GC distribution of *P. pacificum* genome and four other diatom genomes.


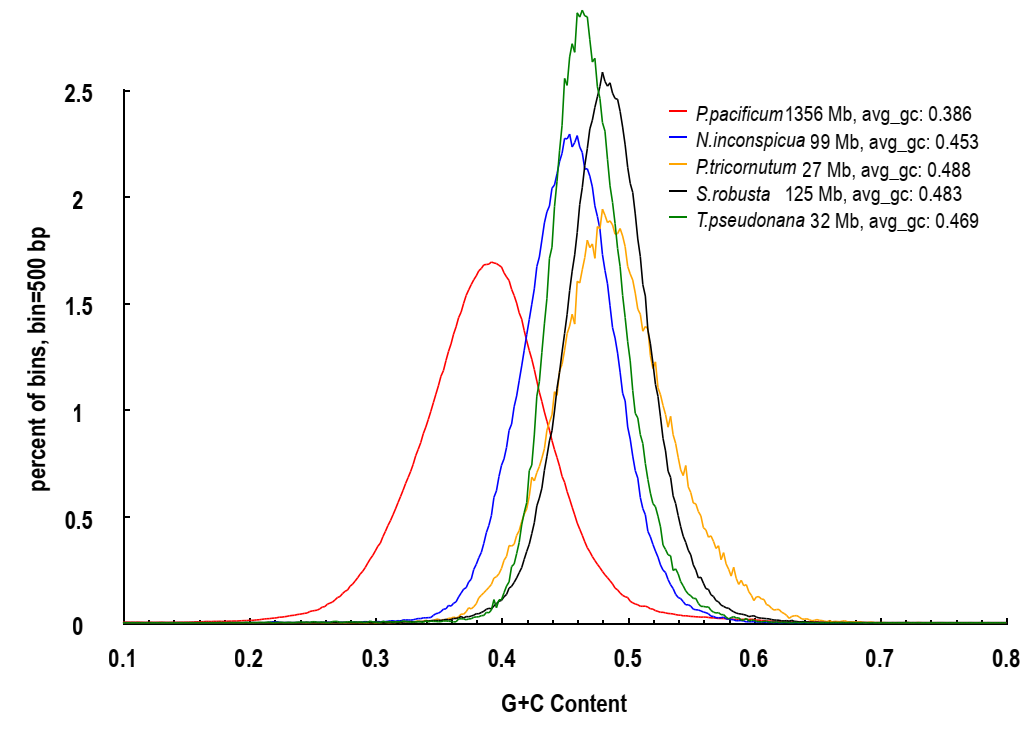


# Figure S5. The repeat distribution of *P. pacificum* genome.


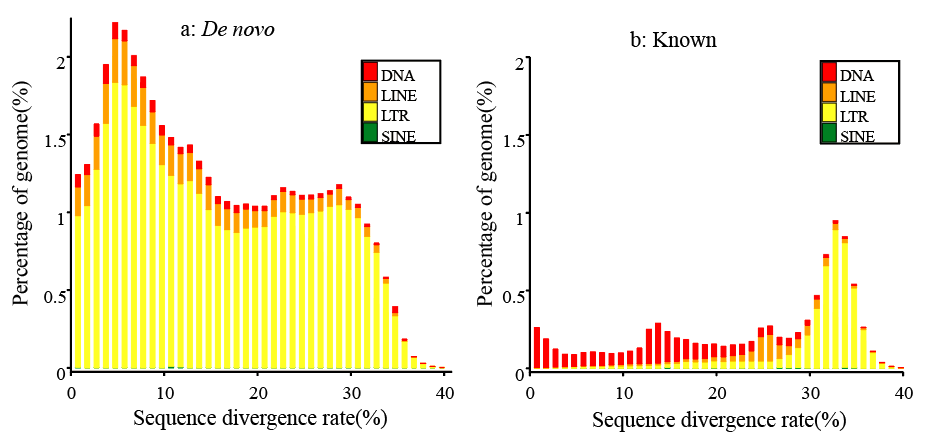


a) repeat annotation with De novo methods. b) repeat with known database.

# Figure S6. The gene features distribution of *P. pacificum* genome and four other diatom genomes.


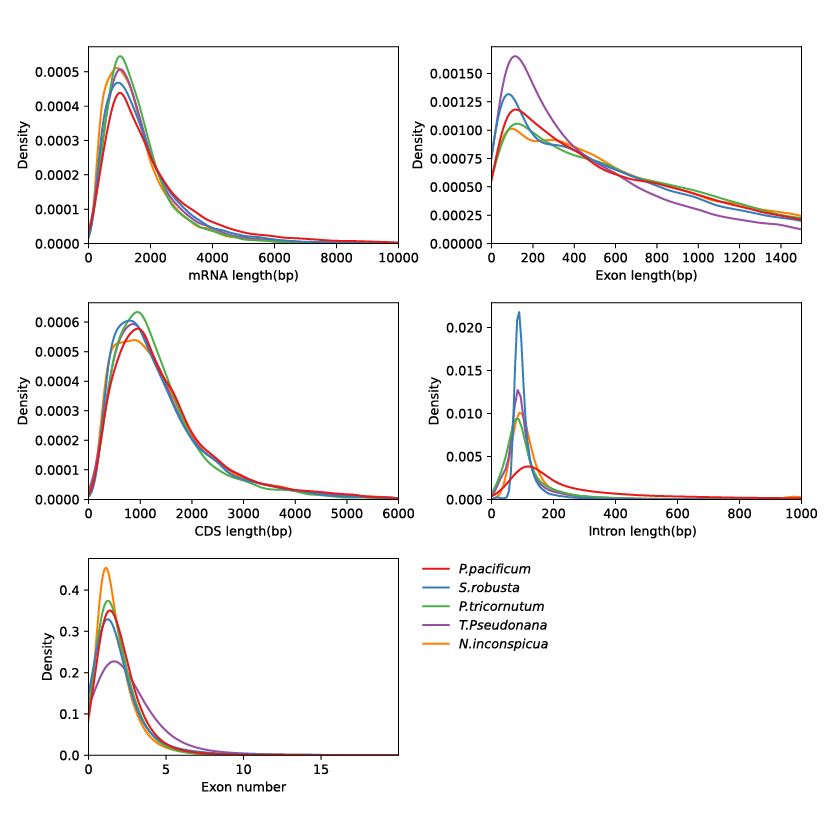


# Figure S7. Gene Ontology (GO) annotation of *P. pacificum*


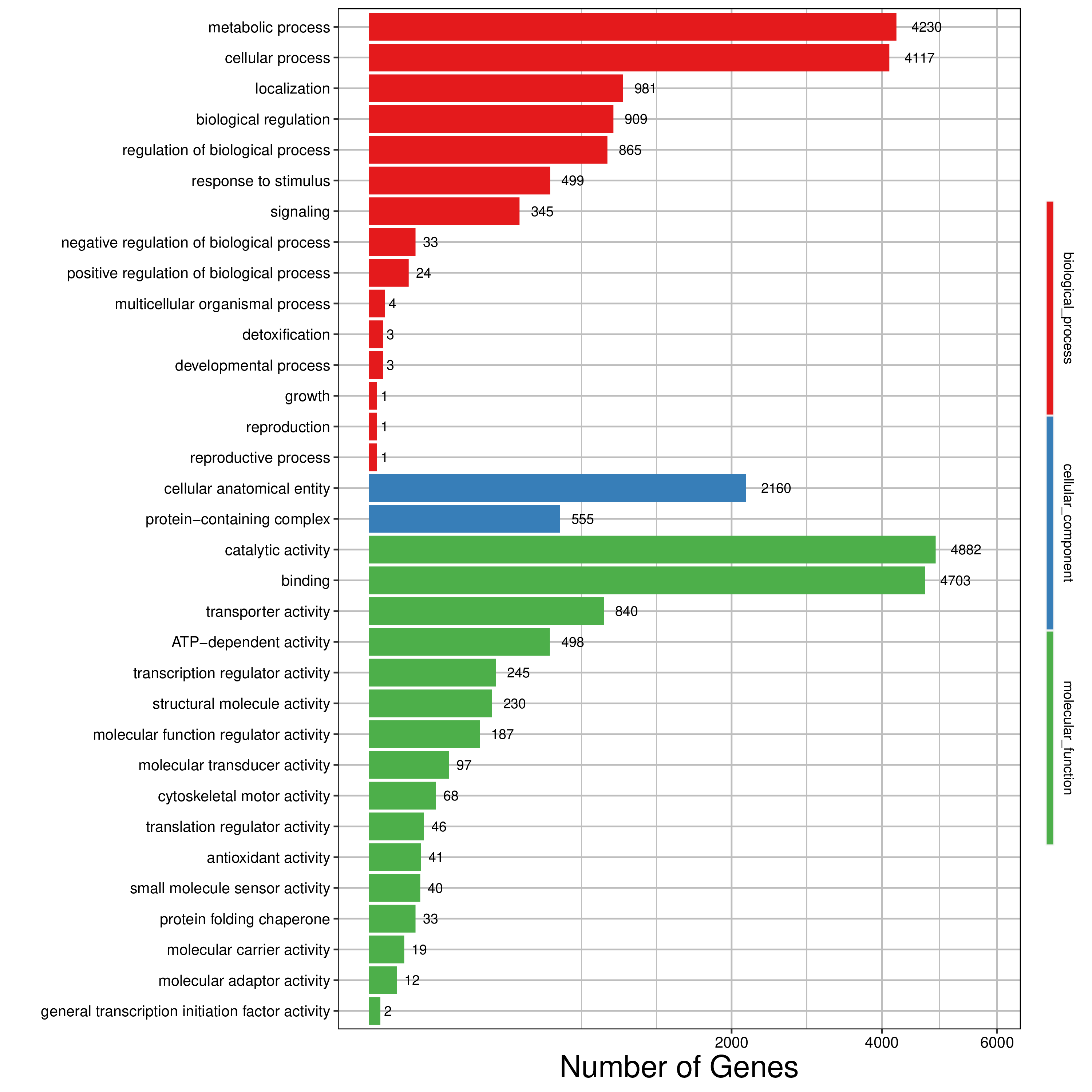


# Figure S8. Kyoto Encyclopedia of Genes and Genomes (KEGG) annotation of *P. pacificum*


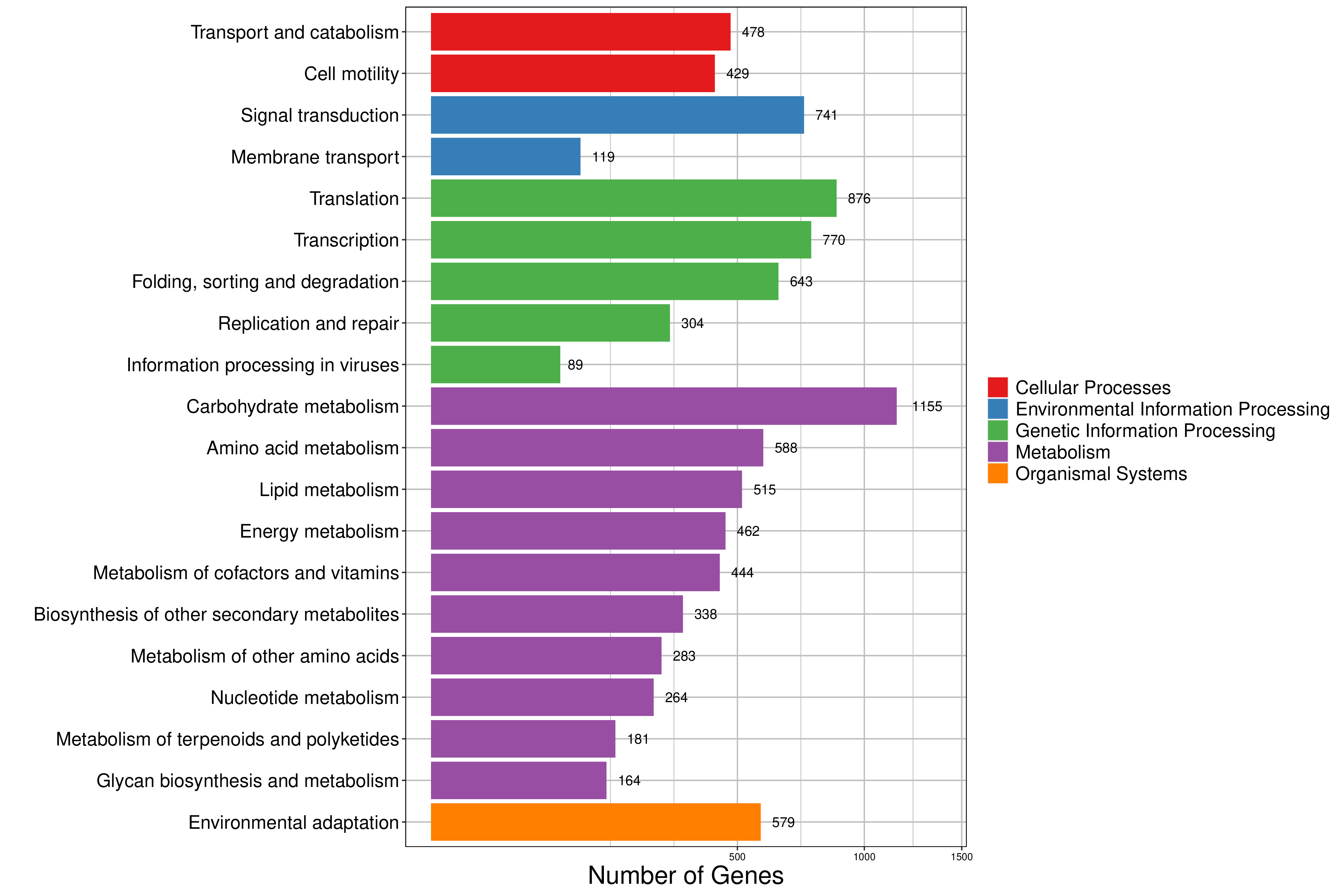


# Figure S9. Venn diagram of functional annotation of *P. pacificum* in five databases.


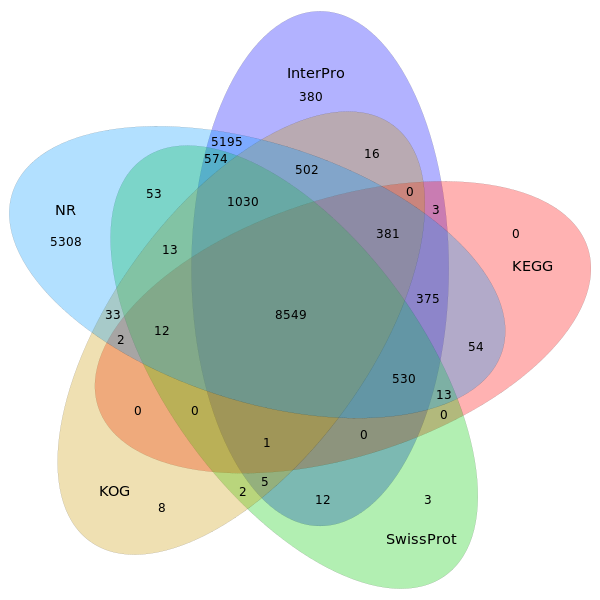


# Figure S10. PCA analysis of Top 10% InterPro Domains


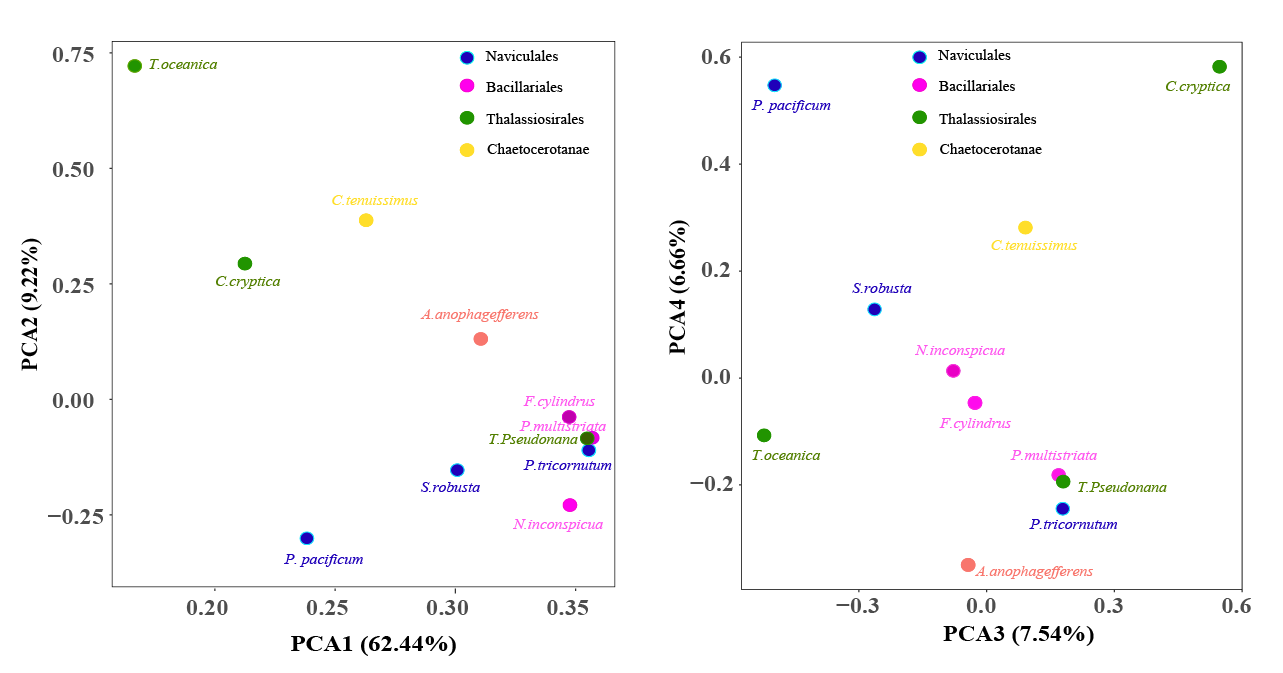


# Figure S11. Heatmap analysis of Top 10% conserved InterPro Domains


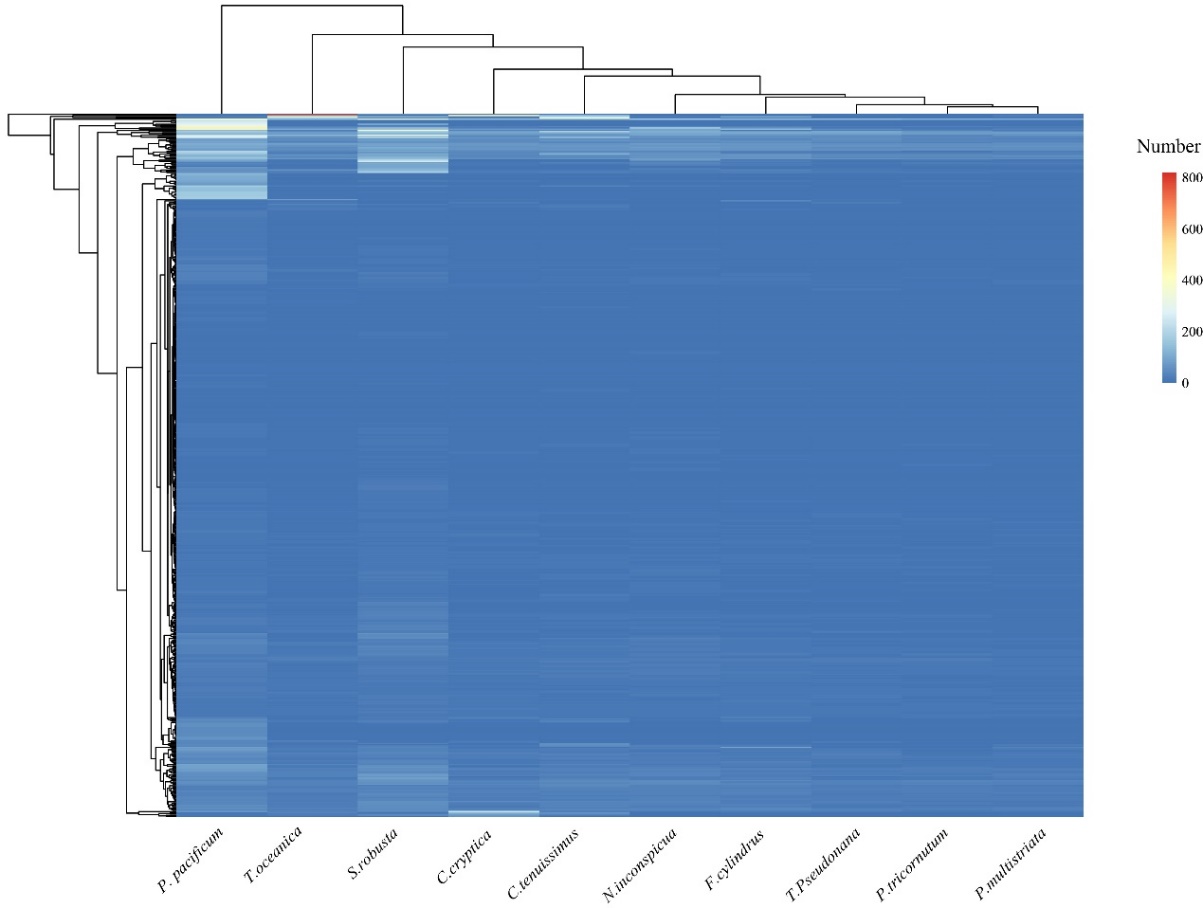


The top 10% is the most abundant annotated domains in all 10 genomes, The top 10% domains are compared with among the 10 genomes.

# Figure S12. Heatmap of Top 10 InterPro Domains


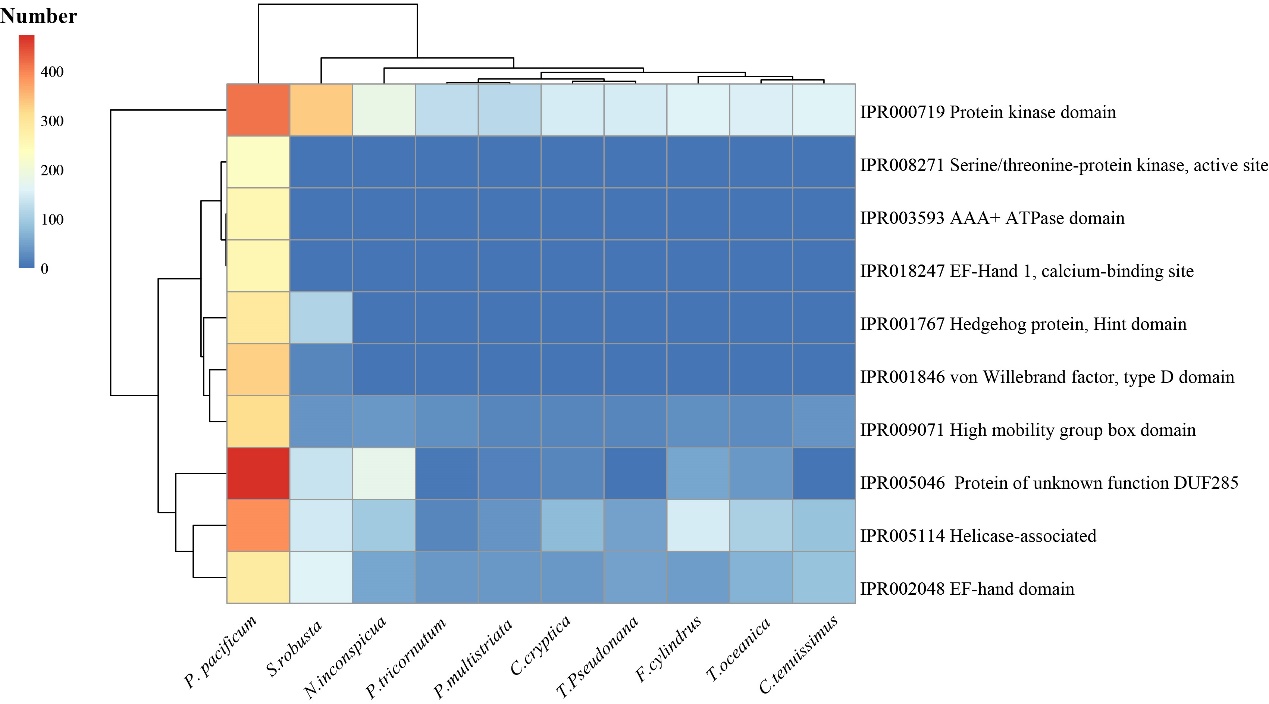


The top 10 InterPro Domains in here is the ten most abundant InterPro-annotated domains in *P. pacificum*

# Figure S13. Comparison of gene families among *P. pacificum* and 10 other algal species


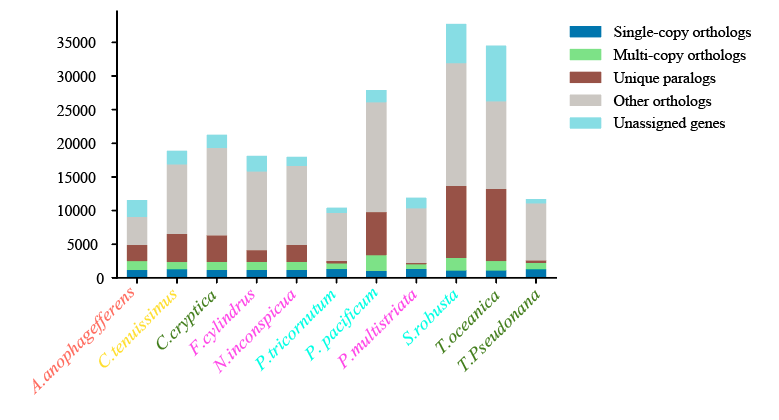


# Figure S14. A phylogenetic tree based on 371 single-copy orthogroups and 1,626 common orthogroups of eleven algal species.


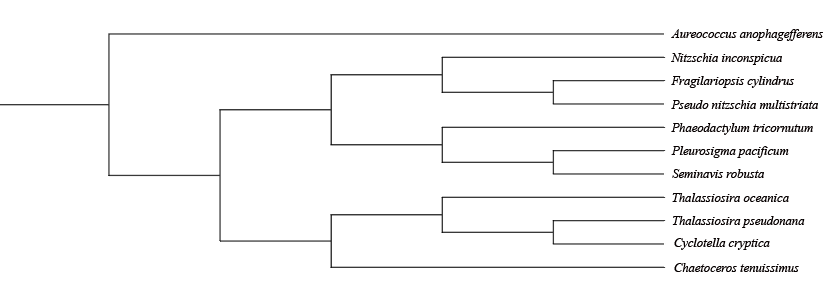


All the support value is 1.

# Figure S15. Heat map of transcription factors (TFs) of 10 diatom genomes.


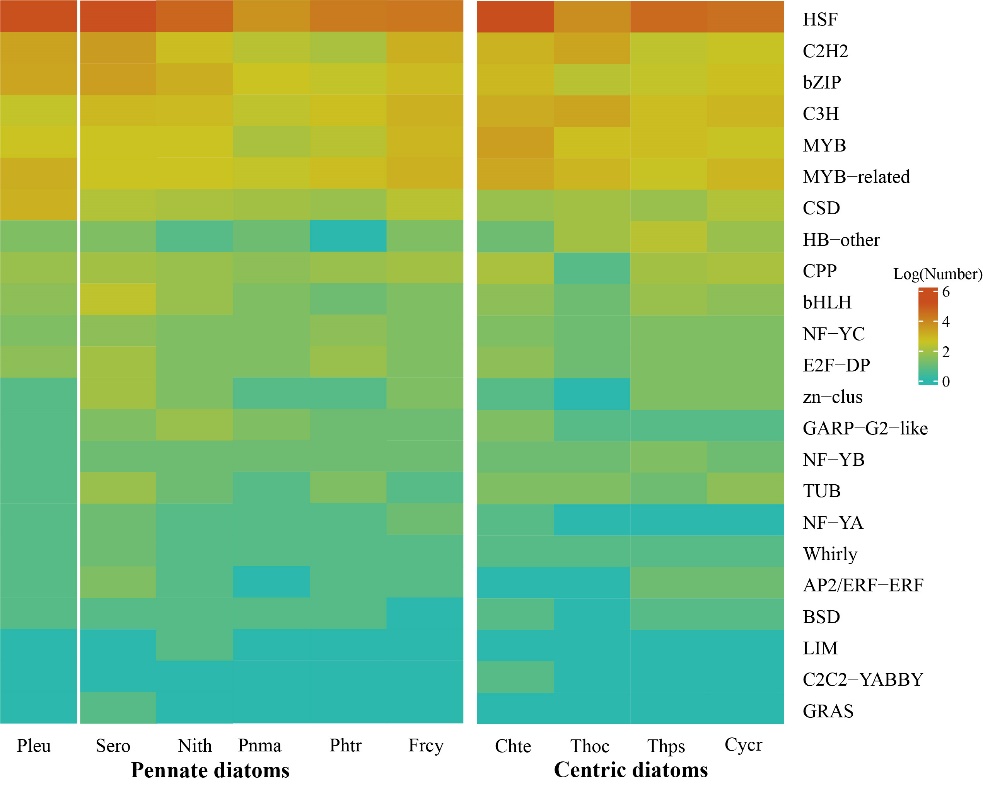


The centric diatoms （*Chaetoceros tenuissimus*: Chte; *Cyclotella cryptica*: Cycr; *Thalassiosira oceanica*: Thoc *Thalassiosira pseudonana*: Thps）; The pennate diatoms (*Fragilariopsis cylindrus*: Frcy; *Nitzschia inconspicua*: Nith; *Phaeodactylum tricornutum*: Phtr; *Pleurosigma pacificum*: Pleu; *Pseudo nitzschia multistriata*: Pnma; *Seminavis robusta*: Sero).

# Figure S16. Heat map of transcription regulators (TRs) of 10 diatom genomes.


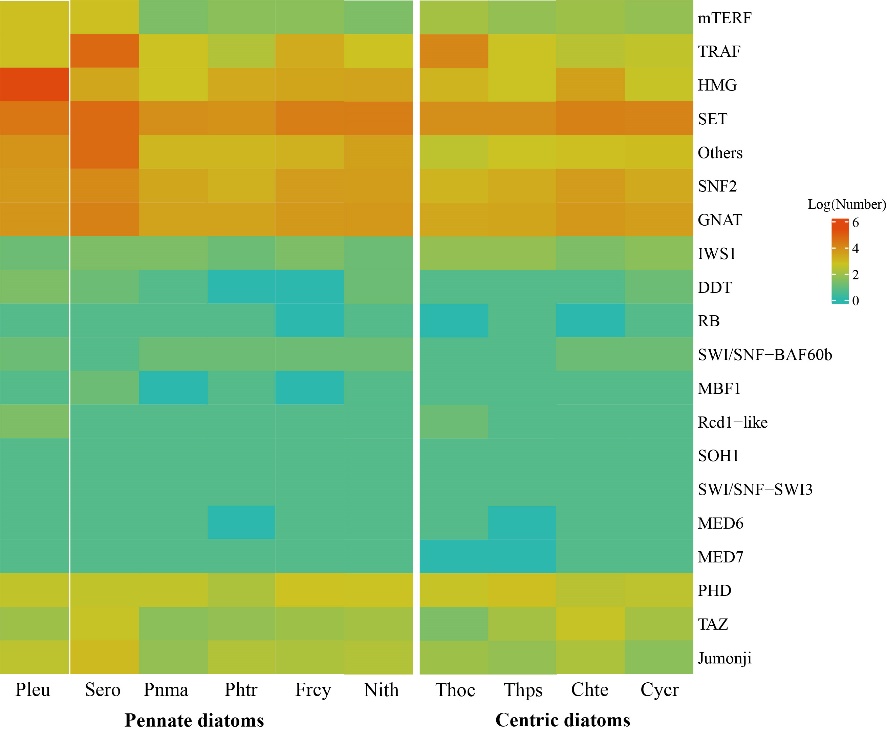


The centric diatoms （*Chaetoceros tenuissimus*: Chte; *Cyclotella cryptica*: Cycr; *Thalassiosira oceanica*: Thoc *Thalassiosira pseudonana*: Thps）; The pennate diatoms (*Fragilariopsis cylindrus*: Frcy; *Nitzschia inconspicua*: Nith; *Phaeodactylum tricornutum*: Phtr; *Pleurosigma pacificum*: Pleu; *Pseudo nitzschia multistriata*: Pnma; *Seminavis robusta*: Sero).

# Figure S17. Heat map of protein kinases (PKs) of 10 diatom genomes.


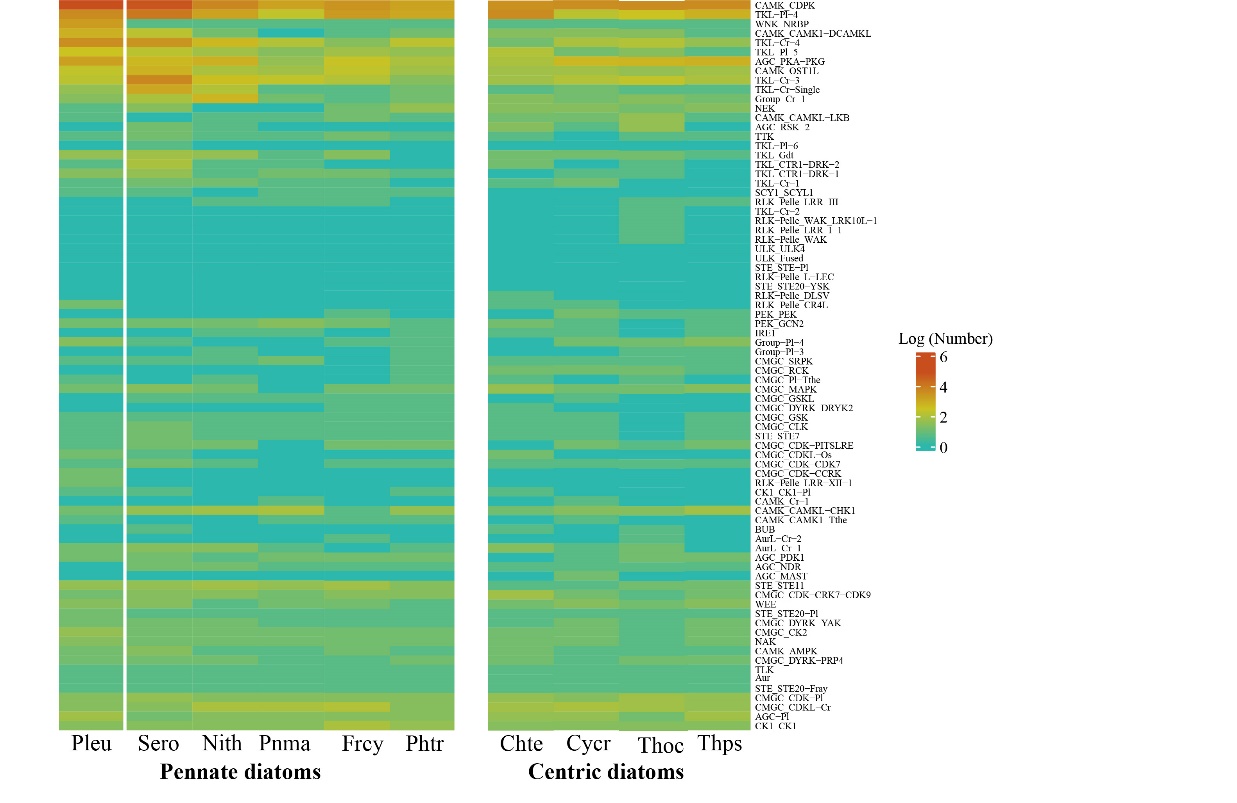


(*The left is six pennate diatoms and right is four centric diatoms, *Chaetoceros tenuissimus*: Chte; *Cyclotella cryptica*: Cycr; *Thalassiosira oceanica*: Thoc; *Thalassiosira pseudonana*: Thps; *Fragilariopsis cylindrus*: Frcy; *Nitzschia inconspicua*: Nith; *Phaeodactylum tricornutum*: Phtr; *Pleurosigma pacificum*: Pleu; *Pseudo nitzschia multistriata*: Pnma; *Seminavis robusta*: Sero).

# Figure S18. KEGG enrichment of expanded genes in *P. pacificum* and *S. robusta*.


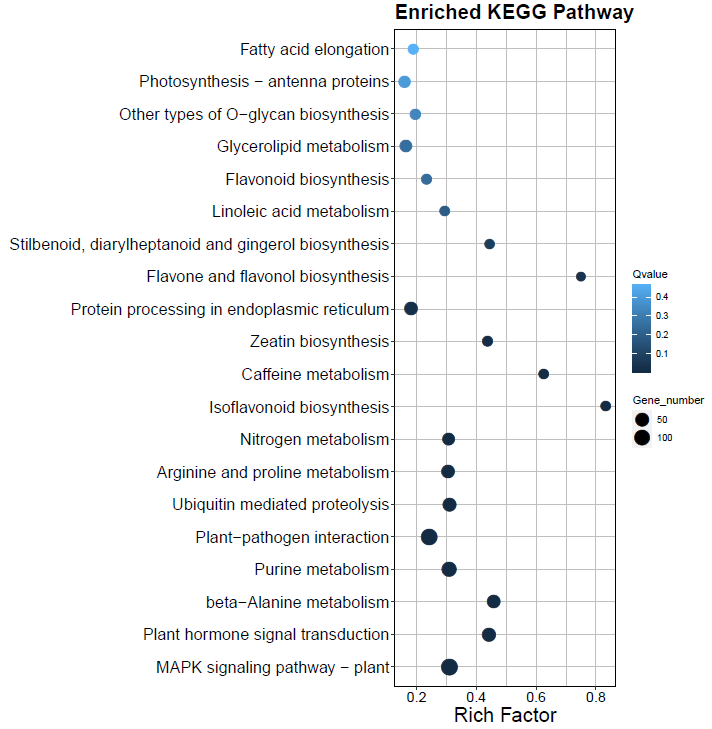


# Tables S1. Sequencing data statistics of *Pleurosigma pacificum* genome with DNBSeq platform.

| Sample | Data | Total number of bases | Q20 | Q30 |
| --- | --- | --- | --- | --- |
| *Pleurosigma pacificum* | Raw reads(fq1) | 26,389,839,450 | 97.76% | 93.17% |
|  | Clean reads(fq1) | 26,313,658,800 | 97.79% | 93.24% |
|  | Raw reads(fq2) | 26,389,839,450 | 97.26% | 93.35% |
|  | Clean reads(fq2) | 26,313,658,800 | 97.24% | 93.43% |
|  | Clean fq1 and fq2 | 52,627,317,600 | 97.52% | 93.33% |

# Table S2. Sequencing data statistics of two strains of *P. pacificum* genomes with PacBio Sequel II platform.

| **HiFi Cell** | **Reads Number** | **Total Length** | **Min Length (reads)** | **Average Length** | **Max Length (reads)** | **Length N50 (reads)** |
| --- | --- | --- | --- | --- | --- | --- |
| Cell1 | 2,342,140 | 37,087,545,380 | 525 | 15,835 | 49,702 | 16,150 |
| Cell2 | 1,955,150 | 28,621,679,377 | 501 | 14,639 | 43,310 | 14,860 |
| **Total** | **4,297,290** | **65,709,224,757** |  |  |  |  |

# Table S3. The initial assembly statistics of *P. pacificum* genomes.

|  | **Contig (bp)** | **Number** |
| --- | --- | --- |
| max length | 13,531,230 |  |
| N10 | 9,247,709 | 13 |
| N20 | 6,354,478 | 32 |
| N30 | 5,187,253 | 56 |
| N40 | 3,942,181 | 87 |
| N50 | 3,191,436 | 127 |
| N60 | 2,644,686 | 176 |
| N70 | 2,002,265 | 237 |
| N80 | 1,459,809 | 320 |
| N90 | 796,651 | 450 |
| Total length | 1,403,309,249 |  |
| number>2000bp | 1,837 |  |
| GC rate | 0.386 |  |

Note: the assembled genomes contained the nuclear genomes, organelle genomes and contamination sequences.

# Table S4. The genome assembly statistics of *P. pacificum* genomes.

|  | Contig (V1) | | Contig (V2) | |
| --- | --- | --- | --- | --- |
|  | length(bp) | number | length(bp) | number |
| Max length | 13,531,230 |  | 13,531,230 |  |
| N10 | 9,247,709 | 13 | 9,247,709 | 13 |
| N20 | 6,487,157 | 31 | 6,522,904 | 30 |
| N30 | 5,233,968 | 54 | 5,233,968 | 54 |
| N40 | 4,014,712 | 84 | 4,051,659 | 83 |
| N50 | 3,228,431 | 122 | 3,228,431 | 121 |
| N60 | 2,718,513 | 169 | 2,725,210 | 167 |
| N70 | 2,065,486 | 225 | 2,079,716 | 223 |
| N80 | 1,533,083 | 302 | 1,533,503 | 300 |
| N90 | 937,448 | 415 | 950,498 | 411 |
| Total length | 1,364,772,876 |  | 1,356,789,042 |  |
| number>2000bp |  | 891 |  | 821 |
| GC rate | 0.386 |  | 0.386 |  |

V1: Decontamination with 90% identity over 10% coverage (but at least 500 bp)

V2: Decontamination with 5% coverage based on V1.

# Table S5. BUSCO analysis result of *P. pacificum* genome and evaluation.

| *P. pacificum* | **Type** | **Complete BUSCOs (C)** | **Complete and single-copy BUSCOs (S)** | **Complete and duplicated BUSCOs (D)** | **Fragmented BUSCOs (F)** | **Missing BUSCOs (M)** | **Total BUSCO groups searched** |
| --- | --- | --- | --- | --- | --- | --- | --- |
| Genome | BUSCO (number) | 202 | 188 | 14 | 16 | 37 | 255 |
|  | BUSCO (percentage %) | 79.2 | 73.7 | 5.5 | 6.3 | 14.5 | 100 |
| Gene | BUSCO (number) | 192 | 176 | 16 | 21 | 42 | 255 |
|  | Gene BUSCO (percentage %) | 75.3 | 69.0 | 6.3 | 8.2 | 16.5 | 100 |

# Table S6. Repeat content in the assembled *P. pacificum* nuclear genomes.

|  | **Repeat Size** | **% of genome** |
| --- | --- | --- |
| TRF | 82,879,771 | 6.11 |
| **Repeatmasker** | 91,195,699 | 6.72 |
| **Proteinmask** | 206,705,631 | 15.23 |
| RepeatMasker ***De novo*** | 760,520,361 | 56.05 |
| **Total** | 817,161,530 | 60.23 |

# Table S7. Transposable elements content in the assembled *P. pacificum* nuclear genomes.

|  | **Repbase TEs** |  | **TE protiens** |  | **De novo** |  | **Combined TEs** | |
| --- | --- | --- | --- | --- | --- | --- | --- | --- |
| **Type** | **Length (Bp)** | **% in genome** | **Length (Bp)** | **% in genome** | **Length (Bp)** | **% in genome** | **Length (Bp)** | **% in genome** |
| DNA | 25,213,150 | 1.86 | 201,351 | 0.01 | 30,066,057 | 2.22 | 54,554,389 | 4.02 |
| LINE | 12,504,581 | 0.92 | 36,921,219 | 2.72 | 77,752,847 | 5.73 | 108,976,272 | 8.03 |
| SINE | 118,818 | 0.01 | 0 | 0.00 | 190,776 | 0.01 | 309,594 | 0.02 |
| LTR | 63,430,565 | 4.68 | 169,586,844 | 12.50 | 649,536,848 | 47.87 | 670,928,977 | 49.45 |
| Other | 9214 | 0.00 | 0 | 0.00 | 0 | 0.00 | 9214 | 0.00 |
| Unknown | 0 | 0.00 | 0 | 0.00 | 4,652,113 | 0.34 | 4,652,113 | 0.34 |
| Total | 91,195,699 | 6.72 | 206,705,631 | 15.23 | 749,707,538 | 55.26 | 782,166,330 | 57.65 |

Note: Repbase TEs: the result of *RepeatMasker* based on Repbase; TE proteins: the result of *RepeatProteinMask* based on Repbase; *De novo*: Result of *RepeatMasker* by using library predicted through *De novo*; Total: combine the results of Repbase TEs, TE proteins and *De novo* with removing the over-lap.

# Table S8. Detailed classification of repeated sequences in *P. pacificum* genome.

| **Type** | | **Length(bp)** | **% of genome** |
| --- | --- | --- | --- |
| **Retro** | LTR/Copia | 522,829,950 | 38.53 |
| **(Retrotransposons)** | LTR/Gypsy | 91,490,759 | 6.74 |
|  | LTR/Other | 79,225,198 | 5.84 |
|  | SINE | 309,594 | 0.02 |
|  | LINE | 108,976,272 | 8.03 |
|  | Other | 0 | 0.00 |
| **DNA (DNA transposons)** | EnSpm | 10,408,884 | 0.77 |
|  | Harbinger | 2,071,384 | 0.15 |
|  | hAT | 6,678,700 | 0.49 |
|  | Helitron | 6,612,871 | 0.49 |
|  | Mariner | 256,573 | 0.02 |
|  | MuDR | 4,171,543 | 0.31 |
|  | P | 247,167 | 0.02 |
|  | Other | 29,181,183 | 2.15 |
| **Other** | - | 11,308,817 | 0.83 |
| **Unknown** | - | 4,652,113 | 0.34 |
| **Total** | - | 792,979,153 | 58.45 |

# Table S9. Statistics of genome elements in 10 diatom genomes.

| **Species** | **Genome size** | **Genome GC** | **Number of genes** | **CDS length** | **CDS GC** | **Repeat (%)** |
| --- | --- | --- | --- | --- | --- | --- |
| *Chaetoceros tenuissimus* | 41,001,288 | 38.9% | 18,869 | 28,654,736 | 40.4% | 12.08 |
| *Cyclotella cryptica* | 171,139,483 | 43.1% | 21,250 | 26,757,525 | 48.2% | 54.00 |
| *Fragilariopsis cylindrus* | 80,540,407 | 38.5% | 18,111 | 22,528,827 | 40.5% | 16.19 |
| *Nitzschia inconspicua* | 99,915,782 | 45.4% | 17,968 | 27,194,238 | 47.6% | 27.39 |
| *Phaeodactylum tricornutum* | 27,450,724 | 48.8% | 10,409 | 14,531,144 | 51.1% | 10.43 |
| *Pleurosigma pacificum* | 1,356,789,042 | 38.6% | 27,408 | 43,648,917 | 45.2% | 60.23 |
| *Pseudo nitzschia multistriata* | 56,765,209 | 46.3% | 11,895 | 19,145,965 | 53.1% | 26.16 |
| *Seminavis robusta* | 125,766,861 | 48.3% | 37,718 | 54,132,365 | 50.0% | 28.93 |
| *Thalassiosira oceanica* | 92,185,637 | 46.9% | 34,500 | 36,780,324 | 56.1% | 36.93 |
| *Thalassiosira pseudonana* | 32,437,365 | 53.3% | 11,674 | 17,492,307 | 47.9% | 6.12 |

# Table S10. Gene function annotation in *P. pacificum*.

| ***P. pacificum*** | **Total** | **Nr-Annotated** | **Swissprot-Annotated** | **KEGG-Annotated** | **KOG-Annotated** | **TrEMBL-Annotated** | **Interpro-Annotated** | **GO-Annotated** | **Overall** |
| --- | --- | --- | --- | --- | --- | --- | --- | --- | --- |
| Number | 27,408 | 22,624 | 10,797 | 9,920 | 10,554 | 14,758 | 17,553 | 9,888 | 23,070 |
| Percentage | 100% | 82.55% | 39.39% | 36.19% | 38.51% | 53.85% | 64.04% | 36.08% | 84.17% |

# Table S11. Number of putative horizontal gene transfer genes in diatoms.

| Name | Number of genes | Bacteria | Archaea | Viruses | Total | HGT Percent |
| --- | --- | --- | --- | --- | --- | --- |
| *C. tenuissimus* | 18,869 | 717 | 126 | 10 | 853 | 4.52% |
| *C. cryptica* | 21,250 | 1127 | 97 | 6 | 1230 | 5.79% |
| *F. cylindrus* | 18,111 | 596 | 83 | 3 | 682 | 3.77% |
| *N. inconspicua* | 17,968 | 665 | 96 | 3 | 764 | 4.25% |
| *P. tricornutum* | 10,409 | 432 | 80 | 1 | 513 | 4.93% |
| *P. pacificum* | 27,408 | 853 | 27 | 2 | 882 | 3.22% |
| *P. multistriata* | 11,895 | 387 | 72 | 0 | 459 | 3.86% |
| *S. robusta* | 37,718 | 1569 | 134 | 21 | 1724 | 4.57% |
| *T. oceanica* | 34,500 | 615 | 111 | 6 | 732 | 2.12% |
| *T. pseudonana* | 11,674 | 568 | 98 | 6 | 672 | 5.76% |
